# Micro-jet formation induced by the interaction of a spherical and toroidal cavitation bubble

**DOI:** 10.1016/j.ultsonch.2024.107185

**Published:** 2024-12-06

**Authors:** Jaka Mur, Alexander Bußmann, Thomas Paula, Stefan Adami, Nikolaus A. Adams, Rok Petkovsek, Claus-Dieter Ohl

**Affiliations:** a Faculty of Natural Sciences, Institute for Physics, Department Soft Matter, Otto-von-Guericke University Magdeburg, Magdeburg, 39106, Germany; b Faculty of Mechanical Engineering, University of Ljubljana, Askerceva 6, Ljubljana, 1000, Slovenia; c Chair of Aerodynamics and Fluid Mechanics, TUM School of Engineering and Design, Technical University of Munich, Garching bei München, 85748, Germany; d Munich Institute of Integrated Materials, Energy and Process Engineering (MEP), Technical University of Munich, Garching bei München, 85748, Germany; e Research Campus STIMULATE, University of Magdeburg, Otto-Hahn-Straße 2, Magdeburg, 39106, Germany

**Keywords:** Cavitation bubble, Toroidal bubble, Micro-jet formation, Secondary cavitation, Amplified rebound

## Abstract

We investigate experimentally and numerically the interaction between a spherical cavitation bubble and a wall-bounded toroidal cavitation bubble. We demonstrate that shock wave focusing following toroidal bubble initiation induces the formation of micro-jets that pierce the spherical bubble in the torus-axis direction away from the surface, strongest in the anti-phase scenario. The velocity of micro-jets is determined by the initial standoff distance of the spherical bubble from the wall and thus from the toroidal bubble, with peak jet velocities approaching 1000m/s. The micro-jets are triggered by the complex interaction between the torus shock wave and the surface of the spherical bubble. Additionally, the formation of secondary cavitation appears to significantly enhance the micro-jets compared to scenarios without secondary cavitation. Following the formation of micro-jets, a subsequent broad jet pierces the spherical bubble, marking the onset of its collapse. After the collapse, we observe an amplified rebound phase resulting in a more than twofold increase of the bubble volume compared to the initial bubble.

## Highlights

• Experiments and simulations of toroidal to spherical cavitation bubble interaction.
• Micro-jet initiation is caused by shock wave focusing from the toroidal bubble.
• Anti-phase bubble interaction induces micro-jets up to 1000m/s.
• Secondary cavitation significantly effects micro-jet initiation process.
• Jetting mechanisms amplify the spherical bubble rebound phase to double initial size.

## Graphical abstract

**Figure:**
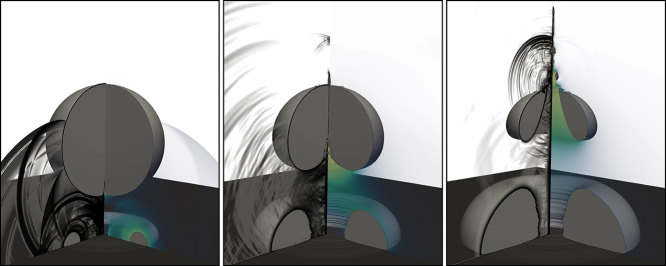

## 1 Introduction

Liquid jets from cavitation bubbles play a crucial role in diverse engineering applications. Uncontrolled, they can cause severe damage to solid surfaces [1]. However, when controlled, stable micro-jets present new opportunities, such as in medical therapies like needle-free injections [2] or for perforating soft-tissue materials [3].

Typically, single cavitation bubble dynamics has been extensively studied both experimentally and numerically. When a cavitation bubble expands and collapses near a flat or complex solid wall driven by a constant far field pressure, complex jetting phenomena occur [4], [5], [6], [7], [8], [9], [10], [11], [12], [13], [14], [15], [16], [17], [18]. High-speed liquid jets, reaching velocities up to O(1000),m/s, have been reported [6], [12], [15], [16], where supersonic needle jets emerge due to a singularity on the symmetry axis caused by radially inward rushing liquid. Such jets are observed for bubbles very close to the solid wall and require an initial expansion phase and flattening of the bubble surface near the wall [12], [16]. Additionally, the wall material plays a significant role, potentially increasing the occurrence of micro-jets at higher standoff distances [17]. For bubbles sufficiently distant from the wall, broader and slower jets driven by pressure differences across the bubble surface are found, with jet velocity and volume strongly dependent on the initial standoff distance [19]. Similar broad jets are observed when bubble collapse is initiated without a prior expansion phase [10], [14].

A gas bubble may also be driven into a jetting flow by exposing it to a shock wave in the absence of boundaries [20], [21], [22], near solid boundaries [23], [24], or elastic surfaces [25], [26] mimicking tissue. Typically, these shock-induced scenarios yield jets, with strengths highly dependent on the post-shock velocity, reaching up to several O(1000)m/s [22], [23]. The jet speed also correlates with the bubble size, with larger bubbles producing higher velocities [20].

Following jet penetration, a toroidal bubble remains, which collapses and undergoes multiple oscillation cycles. The evolution of this toroidal bubble depends on the standoff distance and may lead to attachment to or separated from the wall due to the formation of vortices around the torus bubble [27], [28]. During the toroidal collapse, shock waves are emitted which lead to strong focusing in the torus center yielding much a higher pressures than for the outer diverging shock wave [29], [30], [31], [32]. A recent study of Liu et al. [33] reveals that these focusing effects lead to the formation of a Mach stem inside the torus. Furthermore, such asymmetric collapses can significantly damage the wall through shock-wave focusing on parts of the torus during collapse [1] or may cause surface damage [34], [35].

The complexity of jet formation significantly increases when considering the interaction of multiple cavitation bubbles. To understand this interaction in more detail, tandem arrangements are usually investigated [3], [36], [37], [38], [39], [40], [41], [42], [43], [44]. Studies have shown that the anti-phase scenario, where the second bubble is initiated when the first bubble reaches its maximum size, leads to the fastest jets, ranging from O(100)m/s for millimeter-sized bubbles [38], [40], [43] to O(1000)m/s for smaller bubbles [44]. In these anti-phase scenarios, the jet is induced by the neck collapse of the second bubble penetrating the first bubble [44] and evolves opposite to the growth direction of the second bubble [38], [44].

In this work, we investigate experimentally and numerically the interaction of a wall-bounded toroidal cavitation bubble with a spherical bubble at some distance from the wall. We consider the anti-phase scenario, where the torus is initiated when the spherical bubble reaches its maximum size. We observe the general bubble dynamics during the interaction process, emphasizing jetting mechanisms and bubble rebound. To the best of our knowledge, this is the first systematic investigation of the interaction between a toroidal and a spherical bubble.

The paper is structured as follows. In Section 2, we describe the experimental and numerical setups, focusing on the involved spatial and temporal scales. Section 3 explains the numerical methods used. The dynamics of the interaction are presented in Section 4, including early-stage dynamics (Section 4.1), jet formation and characteristics (Section 4.2), and long-term dynamics with bubble rebound (Section 4.3). Finally, we summarize our findings in Section 5.

## 2 Setup

### 2.1 Experimental setup

**Fig. 1:**
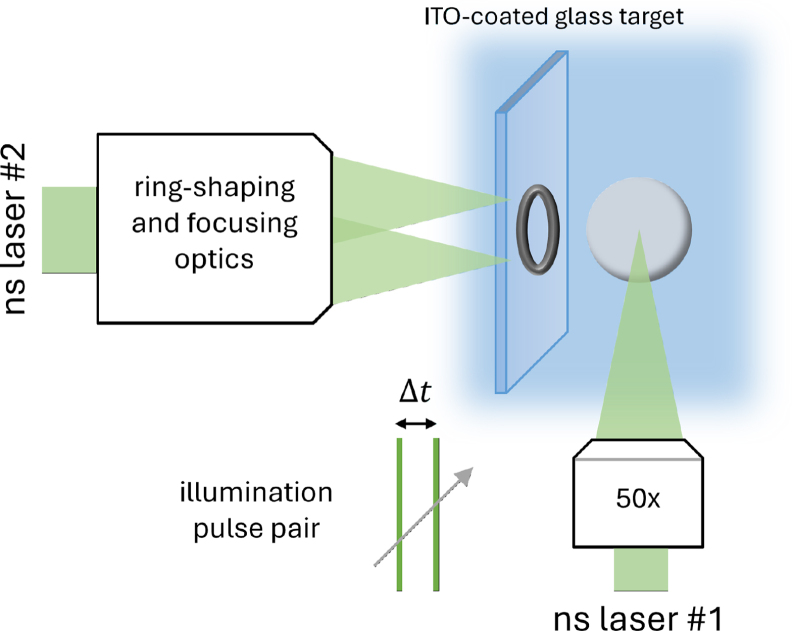
Experimental setup schematic of the main setup features. The first nanosecond laser is focused to a spot, while the second is shaped and focused to the surface of an ITO-coated glass target in water. Ultra-fast camera imaging is done from the side using illumination pulse pairs.

The experimental setup is designed for the observation of two aligned and independent laser-generated cavitation bubbles, the initially generated fully spherical and subsequent semi-toroidal (Fig. 1). The semi-toroidal bubble is generated by a ring-shaped laser focused on a rigid wall that results in an expanding bubble with the shape of a torus cut through its mid-plane, referred in the text as the toroidal bubble. The spherical bubble undergoes dramatic shape changes during its evolution, but is referred to as the spherical bubble within this manuscript. Both cavitation bubbles are excited using green nanosecond lasers, operating at 532 nm wavelength with a pulse duration of 5-6 ns (Litron Nano S and Nano T), coupled to a customized 3D-printed experimental chamber with glass windows on the sides and below. The cuboid cuvette is approximately 28x28x30 mm in size. After inserting the solid boundary used in the experiments, the bubble is generated about 12 mm from the cuvette bottom, 13 mm away from the cuvette walls in all horizontal directions, and at least 15 mm from the free water surface to ensure minimal perturbation of the bubble dynamics compared to a free liquid.

The spherical-bubble excitation laser is focused in a point-like manner using a Mitutoyo 50x long-working distance microscope objective with a numerical aperture of 0.55. The laser energy of the flashlamp pumped Nd:YAG lasers is kept approximately constant by continuously firing the flashlamps and only exiting the Q-switch for the cavitation bubble generation. This allows for achieving a very consistent bubble radius, resulting in bubble lifetimes of $T_{b,\mathrm{cyc}}\approx \text{40}\;\text{µs}$ and maximum bubble radii of $R_{b,\max}\approx \text{210}\;\text{µm}$. The second nanosecond laser is shaped into a ring intensity profile using an axicon lens (Thorlabs AX122-A 2.0 °angle AR coated UVFS axicon) and further focused into water by an aspheric lens (Thorlabs ASL1210 10 mm aspheric lens) built into the wall of the cuvette. To achieve a reliable and repeatable ring-shaped breakdown in water, we used an ITO-coated glass slide as an absorption enhancement target.

Imaging is done using the ultra-high-speed Shimadzu Hyper Vision HPV-X2 camera at 1 MHz and 5 MHz framing rates (resolution of 400 × 250 pixels) for recording of the long-time bubble dynamics and shorter-time bubble interaction. To account for the different scales involved, we used Mitutoyo 5x and 10x objectives for the low and high framing rate, respectively, resulting in 6.3µm/pixel and 3.1µm/pixel resolution on the final images. Ultra-fast imaging is supported by a laser-based pulsed illumination (Ekspla FemtoLux, 515 nm center wavelength) emitting pulse pairs for each ultra-fast-camera frame at an inter-pulse delay of 20 ns and illumination times below 1 ps. Multi-illumination imaging enabled us to resolved the shock wave propagation velocity on a single camera frame close to the collapse site, similar to Reuter et al. [45] and Mur et al. [46]. The alignment of the lasers to position the centers of the toroidal and the spherical bubble is done using an additional fast camera (not shown in Fig. 1) (Fastcam AX-200, Photron).

### 2.2 Numerical setup and initial conditions

**Fig. 2:**
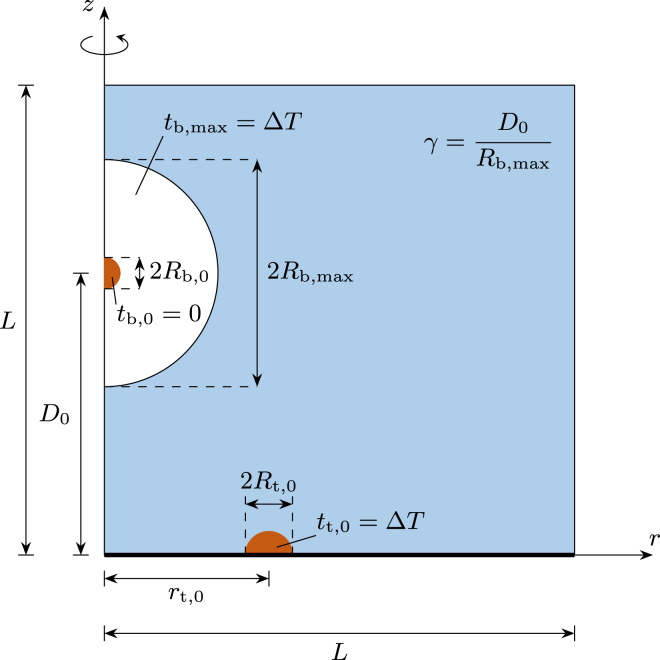
Schematic representation of the three-dimensional axisymmetric setup (r,z) for the toroidal–spherical bubble interaction scenario, where z is the rotation axis. First, a spherical bubble with radius $R_{b,0}$ is initiated in a certain distance $D_{0}$ normal to the wall at $t_{b,0}=\text{0}\;\text{µs}$. The half-toroidal bubble with major radius $r_{t,0}$ and minor radius $R_{t,0}$ is initiated at the wall when the bubble reaches its maximum size $R_{b,\max}$ at t=ΔT≈20µs. In the simulation, on the plane at z=0 a wall boundary condition is used and at r=0 a symmetry boundary condition is employed. The far-field boundaries at z=L and r=L are modeled by a zero-gradient boundary condition. The bubbles are not drawn to scale.

For the interaction of anti-phase bubble pairs, the semi-toroidal bubble is generated at the instant when the spherical bubble reaches its maximum expansion, which follows previous works on bubble pairs [38], [43], [44]. In Fig. 2, a sketch of the anti-phase scenario with all relevant spatial and temporal scales is shown in a three-dimensional axisymmetric setup that is employed for the simulations. For the anti-phase scenario, the spherical bubble is generated at $t=t_{b,0}=0$ at a certain standoff distance $D_{0}$ from the wall. Once the bubble reaches its maximum size, $R_{b,\max}\approx \text{210}\;\text{µm}$, at $t=t_{b,\max}=t_{t,0}=\Delta T\approx \text{20}\;\text{µs}$, the toroidal bubble is initiated.

In the simulations, the spherical bubble is initiated at $t=t_{b,0}$ with an initial radius of $R_{b,0}=\text{8.25}\;\text{µm}$. As the bubble reaches its maximum size of $R_{b,\max}$ at $t=T_{b,\mathrm{cyc}}/2=\Delta T$, the half-toroidal bubble is initiated with a major radius of $r_{t,0}=\text{150}\;\text{µm}$ and a minor radius of $R_{t,0}=\text{5.1}\;\text{µm}$. The initial pressure for both the spherical and the toroidal bubble is fixed to $p_{0}=\text{11 000}\;\text{bar}$ for all variations to drive the expansion into a resting liquid with $p_{\infty }=\text{1}\;\text{atm}$ and $\rho_{\infty }=\text{998}\;\text{kg/m}{}^{3}$. We model both the spherical and toroidal bubbles using a single non-condensable gas. The initial sizes of the spherical and half-toroidal bubble are obtained by matching single cavitation bubble dynamics to experimental observations Appendix B. Therefore, we estimate the initial bubble sizes from the first frames of the experiment and vary them to match the collapse time and the maximum expansion size of the spherical bubble, $R_{\mathrm{b,max}}$, or the maximum outer radius of the half-toroidal bubble $R_{\mathrm{t,o,max}}$.

The computational domain is sufficiently large, $L\approx 350R_{b,\max}$, to minimize numerical reflections of outgoing shock waves. We utilize an adaptive mesh that gradually coarsens towards the far field to optimize computational efficiency. Zero-gradient boundary conditions are applied at all far-field boundaries, while the plane at z=0 is treated as a no-slip wall, and rotational symmetry is enforced at r=0. The smallest cell size is set to $\Delta x_{\min}\approx \text{420}\;\text{nm}$, allowing the initial spherical bubble to be resolved with $N_{b,0}=R_{b,0}/\Delta x_{\min}\approx 20$ cells and the toroidal bubble with $N_{t,0}=R_{t,0}/\Delta x_{\min}\approx 12$ cells. This resolution is sufficient for the used numerical schemes to accurately capture cavitation bubble dynamics Appendix A.

### 2.3 Non-dimensionalization of physical quantities

Throughout this work we use reference parameters marked with an asterisk for nondimensional quantities marked with a hat. Length scales are normalized with the spherical bubble size at maximum expansion

(1) $$l^{*}=R_{b,\max}.$$

Hence, the toroidal bubble geometry is described by the normalized major and minor radius

(2) $${\overset{ˆ}{r}}_{t,0}=r_{t,0}/R_{b,\max}\;\mathrm{and}\;{\overset{ˆ}{R}}_{t,0}=R_{t,0}/R_{b,\max}.$$

The initial standoff distance reads in normalized form

(3) $$\gamma =D_{0}/R_{b,\max},$$

which is adapted from single cavitation bubbles expanding near walls [12], [16] and differs from the case of identical bubble pairs in the free-field, where $\gamma_{\mathrm{ff}}=\gamma /2=D_{0}/\left(2R_{b,\max}\right)$ [38], [44]. The temporal scale is related to the Rayleigh collapse time of a natural collapse of the maximum expanded spherical bubble [47] in an infinite domain and reads

(4) $$T^{*}=R_{b,\max}\sqrt{\rho_{\infty }/\Delta p},$$

Here, $\rho_{\infty }$ is the density of the surrounding liquid and $\Delta p=p_{\infty }-p_{b,\max}$ is the driving pressure difference of the collapse between the surrounding liquid and the spherical bubble at maximum expansion. Velocities and pressures are nondimensionalized with

(5) $$u^{*}=l^{*}/t^{*}=\sqrt{\Delta p/\rho_{\infty }}\;\mathrm{and}\;p^{*}=c_{\infty }\sqrt{\rho_{\infty }\Delta p},$$

where $c_{\infty }$ is the speed of sound of the liquid [14], [19].

## 3 Numerical methods

### 3.1 Governing equations

We employ the open-source code framework ALPACA [48], [49] which solves the compressible Navier–Stokes equations, including viscous effects, based on the RDEMIC multi-phase flow model [50]. The governing equations in three-dimensional form for an arbitrary number of phases read

(6) $$\frac{\partial U_{i}}{\partial t}+\nabla \cdot F_{i}\left(U_{i}\right)=B_{\mathrm{int}}\cdot \nabla \alpha_{i}\;,$$

where $U_{i}=\alpha_{i}\left[1,\rho_{i},\rho_{i}u_{i},\rho_{i}E_{i}\right]$ is the vector of conserved quantities and the convective/viscous fluxes, $F_{i}\left(U_{i}\right)$ are defined as

(7) $$F_{i}\left(U_{i}\right)=\alpha_{i}\left[\begin{array}{c} 0\\ \rho_{i}u_{i}^{T}\\ \rho_{i}u_{i}⊗u_{i}+p_{i}I+T_{i}\\ \left(E_{i}+p_{i}\right)u_{i}^{T}+T_{i}\cdot u_{i} \end{array}\right]\;.$$

In the above equations, $\alpha_{i}$ is the volume fraction, $\rho_{i}$ is the fluid density, $u_{i}=\left[u_{i},v_{i},w_{i}\right]$ the Cartesian velocity vector, $p_{i}$ the thermodynamic pressure, $E_{i}=\rho_{i}e_{i}+1/2\rho_{i}\|u{\|}^{2}$ the total energy combining specific internal energy $e_{i}$ and the kinetic energy, and I the identity matrix. We model viscous effects with a Newtonian stress tensor

(8) $$T_{i}=\mu_{i}\left(\nabla ⊗u_{i}+{\left(\nabla ⊗u_{i}\right)}^{T}\right)-\frac{2}{3}\mu_{i}\nabla \cdot u_{i}I$$

with a constant shear viscosity $\mu_{i}$ and zero bulk viscosity. The subscript i indicates that the governing equations are solved for every phase individually, where the volume fraction $\alpha_{i}$ is bounded by $0\leq \alpha_{i}\leq 1$ under the constraint $\sum_{i}\alpha_{i}=1$. We model exchange terms at fluid–fluid interfaces through the interface source term

(9) $$B_{\mathrm{int}}=\left[\begin{array}{c} -u_{\mathrm{int}}^{T}\\ 0\\ p_{\mathrm{int}}I\\ p_{\mathrm{int}}u_{\mathrm{int}}^{T} \end{array}\right]\;,$$

where $u_{\mathrm{int}}$ is the Cartesian interface velocity and $p_{\mathrm{int}}$ is the interface pressure.

We close the system of equations thermodynamically with the stiffened gas equation of state (EoS)

(10) p(ρ,e)=(κ−1)ρe−κΠ,

where κ is the ratio of specific heats, and Π is the background pressure [51]. Liquid water is modeled with the parameters of the Tait EoS, $\Pi_{l}=\text{3046}\;\text{bar}$, and $\kappa_{l}=7.15$ [52]. For gases, we use the ideal gas EoS with parameters of air, $\Pi_{g}=\text{0}\;\text{bar}$, and $\kappa_{g}=1.4$.

In the governing equations, we account for compressibility effects in both the liquid and gaseous phases, viscous terms, and convective interface effects, as they significantly impact the evolution of cavitation bubble dynamics [53]. Capillary forces, species diffusion, and buoyancy effects are neglected, as their spatial and temporal scales are either much slower or faster and thus have negligible effect on the overall dynamics of the cavitation bubbles.

Phase change effects play an important role in simulating cavitation scenarios, significantly influencing bubble dynamics in the free-field and near solid surfaces [12], [54], [55], [56], [57]. Phase-change effects typically occur during extreme states of the bubble, such as initiation or collapse. Two numerical approaches have been presented to account for phase-change effects. One approach removes mass from the bubble at specific times to effectively reduce the bubble equilibrium radius and enhance bubble collapse [12], [55], [56]. However, this method is calibrated for single, non-interacting bubbles and has no straightforward extension for multiple bubble interactions. Additionally, the timing for a posteriori mass reduction is crucial and is particular unknown for the present scenario. The other approach uses kinetic relations to compute mass transfer across the interface, continuously modeling phase-change effects [54], [57]. This concept relies heavily on accurate material parameters and the validity of kinetic relations over a wide range of thermodynamic states. Given these limitations and uncertainties, we model the bubbles with a single non-condensable gas without phase change, acknowledging potential systematic noted errors at the instant of bubble collapse.

### 3.2 Numerical implementation

We solve the governing equations by a Godunov-type flux-based finite volume approach, integrating Eq. (6) in time with a third-order Runge–Kutta TVD scheme [58]. The fluxes on the cell faces are evaluated by a single- and multiphase HLLC Riemann solver [50] together with a fifth-order WENO characteristic state reconstruction [59] and signal speed estimates following Einfeldt [60]. Viscous stresses are evaluated by a second-order central differencing scheme [61]. All simulations are conducted in a reduced cylindrical space (r,z) introducing singular terms of type 1/r by the divergence operator. We treat these terms as geometric source terms [62], [63].

As stated in the governing equations Eq. (6), the interface of each phase is advected by an individual volume fraction, $\alpha_{i}$, leading to excessive smearing of the interfacial region without any further treatment [50]. We regularize the interface by a linear multidimensional THINC reconstruction, balancing diffusion and compression contributions, with a compression value of β=3.

Due to complex interface and wave dynamics during the evolution and interaction of multiple cavitation bubbles, complex states may arise numerically. Therefore, we employ a pre-integration of the THINC interface [50] and a MOOD-like post-integration step for preserving positivity [64]. We limit the densities within the cavitation bubbles to a minimum of $\rho_{\min}=\text{0.001}\;\text{kg/m}{}^{3}$ to prevent strongly rarefied fluid states.

We employ a block-based multiresolution scheme (MR) to improve computational efficiency, where regions of strong fluid variations are resolved with higher resolutions [65]. We evaluate the smoothness of a field by a wavelet analysis [66]. Interface regions are always maintained on the finest level to capture the interface dynamics accurately, and interface operations are only performed within a narrow band of about eight cells around the interface using a volume fraction cutoff of $\alpha_{i,c}=10^{-9}$. For cells at the edges of the narrow band, we reduce the reconstruction order successively using fifth- [59], third- [67], and first-order WENO schemes.

## 4 Results and discussion

### 4.1 Early stage dynamics of toroidal–spherical bubble interaction

**Fig. 3:**
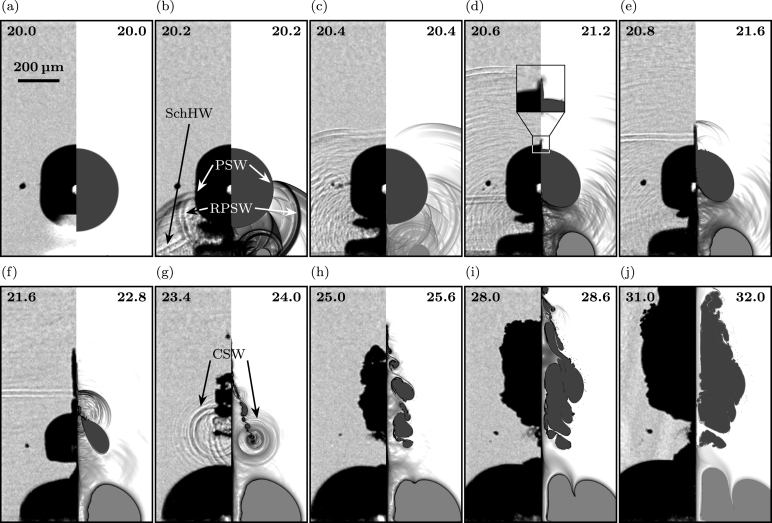
Early stage dynamics comparing simulation and experiment of the anti-phase toroidal–spherical cavitation bubble scenario for a nondimensional standoff distance of γ≈1.5: High-speed side-view dynamics of the experiment (left half of each image) and visualization of numerical Schlieren in the simulation (right half of each image). The experimental shadowgraphs show a bubble projection, while the simulation shows a plane cutting the bubble. Time instants are given in micro seconds at the top of each image, and in (a) a scale bar for all images is shown. In (b) and (g), white and black arrows indicate the position of the initial plasma shock wave (PSW), the reflected plasma shock wave from the spherical bubble (RPSW), the Schmidt head wave (SchHW), and the collapse shock wave (CSW).

**Fig. 4:**
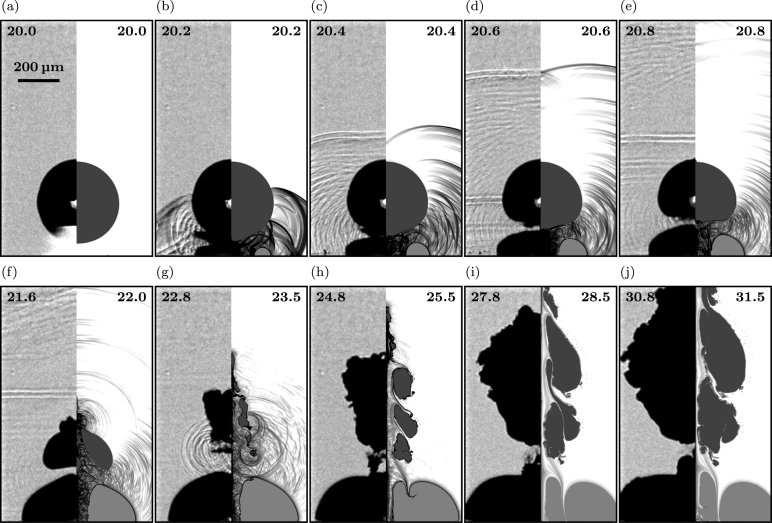
Early stage dynamics comparing simulation and experiment of the anti-phase toroidal–spherical cavitation bubble scenario for a nondimensional standoff distance of γ≈1.2: High-speed side-view dynamics of the experiment (left half of each image) and visualization of numerical Schlieren in the simulation (right half of each image). The experimental shadowgraphs show a bubble projection, while the simulation shows a plane cutting the bubble. Time instants are given in micro seconds at the top of each image, and in (a) a scale bar for all images is shown.

**Fig. 5:**
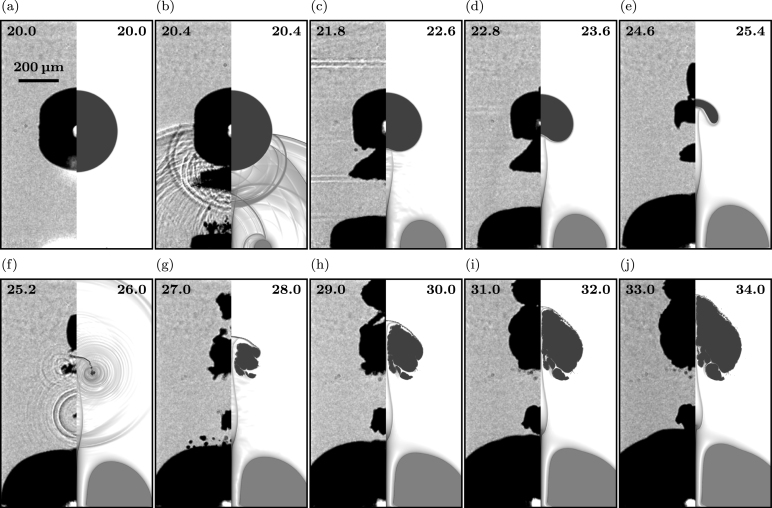
Early stage dynamics comparing simulation and experiment of the anti-phase toroidal–spherical cavitation bubble scenario for a nondimensional standoff distance of γ≈2.7: High-speed side-view dynamics of the experiment (left half of each image) and visualization of numerical Schlieren in the simulation (right half of each image). The experimental shadowgraphs show a bubble projection, while the simulation shows a plane cutting the bubble. Time instants are given in micro seconds at the top of each image, and in (a) a scale bar for all images is shown.

Fig. 3, Fig. 4, Fig. 5 give an overview of the bubble dynamics for the anti-phase toroidal–spherical bubble interaction scenario at nondimensional standoff distances γ≈1.5 (Fig. 3), γ≈1.2 (Fig. 4), and γ≈2.7 (Fig. 5). In addition, Appendix C shows nondimensional standoff distances γ≈1.85 and γ≈3.2. The images compare numerical Schlieren of the simulation (right half of each image) with experimental shadowgraphy (left half of each image). Bubbles are shown in black (experiment) and gray (simulation). Time instants at the top of each image are chosen to show similar stages of the interaction dynamics in experiment and simulation.

For a standoff distance of γ≈1.5 (Fig. 3), we observe that shortly after initiation of the toroidal bubble (Fig. 3a), emitted shock waves from the ring plasma primarily control the bubble dynamics (Fig. 3b-c). The initial plasma shock wave (PSW) travels towards the surface at the lower pole of the spherical bubble, reflects, and undergoes multiple subsequent reflections between the spherical and toroidal bubble. In the experiment, a Schmidt head wave (SchHW) is also visible, originating from the reflection of the PSW at the wall and its transmission into the solid with a speed of sound higher than the liquid [25], [68], [69] (Fig. 3b). This surface wave is not present in the simulation as we neglect the (hyper)elastic solid and initially attach the toroidal bubble to the wall. Due to the shock-dominated flow, in the experiment, a secondary cavitation cloud forms between the spherical and the toroidal bubble (Fig. 3b-c), a phenomenon not captured in the simulation due to the absence of an explicit nucleation model for secondary cavitation. In Fig. 3d, a micro-jet appears on the upper side of the spherical bubble, traveling upwards in wall-normal direction. In the simulation, the micro-jet appears slightly later than in the experiment and is less elongated (Fig. 3d-f). During the ejection of the micro-jet, a subsequent broad jet forms from the lower pole of the bubble and pierces it on the upper bubble side (Fig. 3f). After jet piercing, the spherical bubble collapses, and initiates bubble rebound (Fig. 3g). During the rebound phase, the spherical bubble elongates upwards and amplifies in size (Fig. 3h-j). The secondary cavitation cloud remains visible throughout the entire jetting process (Fig. 3b-f) and disappears after the collapse of the spherical bubble (Fig. 3g). During these early stages of the dynamics, the wall-bounded toroidal bubble grows in width and height, similarly to its growth behavior without presence of a spherical bubble. Differences become apparent after the collapse of the pierced spherical bubble (Fig. 3g). Due to the emitted collapse shock wave (CSW), a constriction forms within the wall-bounded toroidal bubble following the impact of the CSW on the top of the toroidal bubble surface (Fig. 3h). This constriction moves downward, creating a cusp in the torus center, visible in both the experiment and simulation (Fig. 3i-j).

When we compare the dynamics with the scenario of a lower nondimensional standoff distance, γ≈1.2 (Fig. 4), we observe identical initial dynamics (Fig. 4a-c). Multiple reflections of the initial plasma shock wave on the spherical and toroidal bubble surface lead to complex wave formations, including a Schmidt head wave in the experiment (Fig. 4b). Again, a secondary cavitation cloud forms between the toroidal and spherical bubble; however, it vanishes significantly earlier (Fig. 4b-c). Compared to the larger standoff distance, the interface deformation and wave dynamics on the lower side of the bubble are more complex and no narrow and elongated stable micro-jet is visible on the upper side (Fig. 4d-e). The broad jet pierces the spherical bubble earlier than for the larger standoff distance and initiates its collapse (Fig. 4f-g). After the collapse, spherical bubble rebound expands and amplifies it in width and height (Fig. 4h-j). For the wall-bounded toroidal bubble, we again find the formation of a constriction within the torus due to the impact of the CSW on the upper toroidal bubble surface (Fig. 4h-j). However, the shock wave impacts the surface closer to the symmetry axis, forming a sharper cusp in the torus center.

For significantly larger standoff distances, γ≈2.7 (Fig. 5), the initial dynamics after toroidal bubble initiation reveal similar complex wave interactions following the impact of the initial plasma shock wave on the spherical bubble surface, accompanied by the formation of a secondary cavitation cloud (Fig. 5b). In contrast to the lowest standoff distance, γ≈1.2, a micro-jet forms in the experiment on the upper bubble side. However, it appears shorter and thicker than for the intermediate standoff distance, γ≈1.5 (Fig. 5d-f). Simulations do not show the micro-jet forming only the subsequent broad jet that pierces the bubble and initiates collapse of the remaining spherical bubble (Fig. 5e-f). Following the collapse, the spherical bubble rebounds (Fig. 5g-j). However, the amplification during the rebound stages is significantly smaller at the larger standoff distance. Once the broad jet pierces the upper side of the bubble, a portion of the bubble separates from the main bubble and expands slightly above it. Due to the amplified rebound of the spherical bubble, both parts eventually merge again (Fig. 5g-j). In the simulations, only the rebound of the main bubble is observed.

Another characteristic of this larger standoff distance is the evolution of the secondary cavitation cloud, initiated by the complex wave dynamics after the initiation of the wall-bounded toroidal bubble. The cloud is significantly larger and persists for a longer time period. After nucleation, parts of the secondary cavitation cloud are sucked into the spherical bubble due to the extended duration of jet formation (Fig. 5c-d). Eventually, only a portion of the original cloud remains between the toroidal and spherical bubble (Fig. 5e). This remaining part eventually collapses (Fig. 5f) and rebounds independently of the spherical bubble (Fig. 5g-h). It finally merges with the growing wall-bounded toroidal bubble.

When examining the evolution of the wall-bounded toroidal bubble, we observe that, unlike at smaller γ values, its growth is not affected by the shock wave of the collapsing spherical bubble (Fig. 5f-j). This behavior is caused by the increased distance of the spherical bubble from the toroidal and the corresponding dampening of the shock wave before it impacts the toroidal bubble surface.

### 4.2 Jet formation and characteristics

**Fig. 6:**
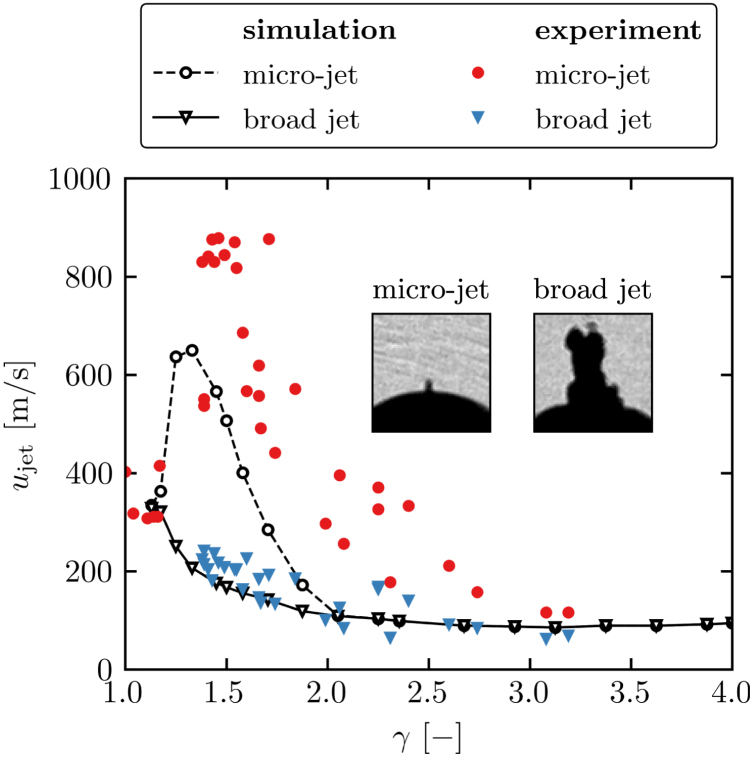
Jet velocity of the initial micro-jet and subsequent broad jet over the nondimensional standoff distance for the simulation and the experiment. The appearance of the jets is visualized in the insets.

During early stage dynamics, we observe the presence of a micro-jet on the upper side of the spherical cavitation bubble that forms on the lower bubble side. Subsequently, a broad jet develops from the lower side of the bubble. In Fig. 6, the jet velocity is plotted over the nondimensional standoff distance. Experimentally, we have estimated the micro-jet velocity from the time difference between the instance the micro-jet appears on the upper bubble side and the instance the torus shock wave impacts on the lower bubble side, and computing the traveled distance from the lower side of the spherical bubble to the upper pole (left inset in Fig. 6). More complex is the measurement of the broad jet, where we approximate the time instant when the micro-jet separates from the spherical bubble or a subsequent broad jet develops on the upper bubble side (right inset in Fig. 6). Numerically, the two jets can clearly be identified through the formation of pressure waves at jet piercing.

For very large standoff distances, in the experiment, slower and thicker jets are observed (e.g., Fig. 5d-e) where velocities around 100m/s are reached. When the standoff distance decreases, the jet velocity increases non-linearly and reaches peak values around 900m/s at γ≈1.5. Then, the higher jet velocity translates into a thinner and elongated micro-jet. The jet velocity decreases rapidly for even smaller standoff distances and reaches around 300m/s at γ≈1. When examining the subsequent broad jet, we observe a different trend. For large standoff distances, the jet velocity is around 65m/s and continuously increases for smaller standoff distances. It reaches its maximum of 300m/s at γ≈1, making the two jet types hardly distinguishable.

In the simulation, similar trends are observed; however, quantitatively, we find differences. No micro-jet is observed for large standoff distances, and only broader and slower jets around 80–90m/s are found. This velocity remains independent of the standoff distance in the range γ∈[2,4]. When the distance towards the wall decreases, the subsequent broad jet velocity increases and reaches a maximum of 300m/s at γ≈1. In the simulations, faster micro-jets start to form at around γ≈2, and the jet velocity increases non-linearly towards a peak of 650m/s at γ≈1.4. For smaller distances, the jet velocity quickly reduces towards 300m/s at γ≈1.

**Fig. 7:**
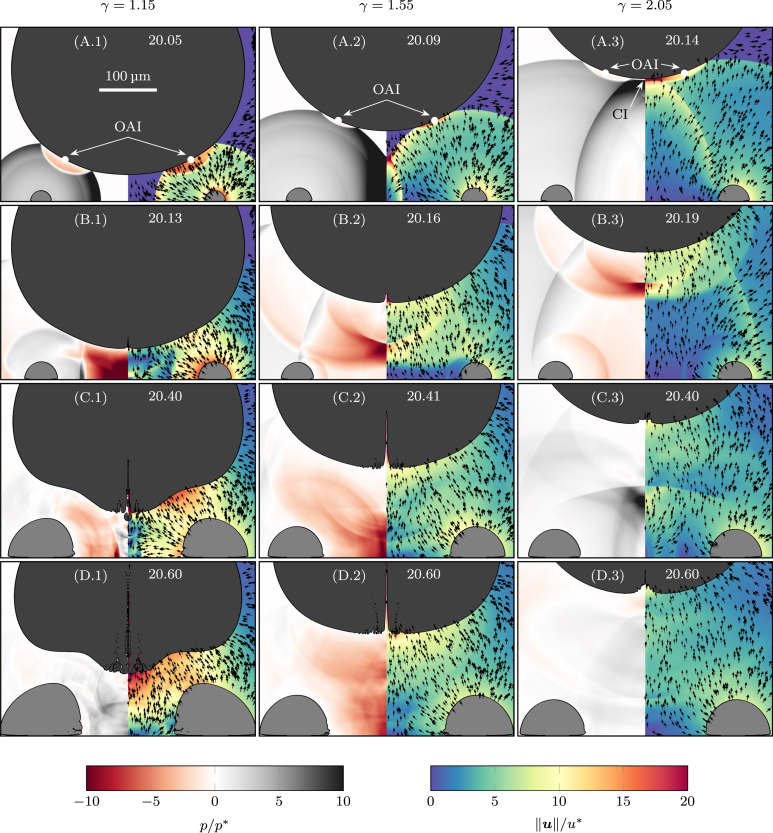
Visualization of the pressure and velocity field in the first 600ns after initiating the toroidal bubble: Shown are three different standoff distances, γ≈1.15 (A-D.1), γ≈1.55 (A-D.2), and γ≈2.05 (A-D.3) at four different time instants (A)-(D). In the left half of each image, we visualize the normalized pressure field, $p/p^{*}$. The right half shows the normalized velocity magnitude, $\|u\|/u^{*}$, overlaid with unscaled velocity vectors. The color bars for each image are given at the bottom, time instants are shown at the top of each image in microseconds, and a scale bar for all images is given in (A.1). In (A.1-3) the off-axis impact (OAI) and center impact (CI) of the PSW is marked.

To understand the formation process of the faster micro-jets, we show in Fig. 7 the pressure and velocity fields at the initial stages after initiating the wall-bounded toroidal bubble. Three different nondimensional standoff distances, γ≈{1.15,1.55,2.05}, are given at four time instants.

After initiating the toroidal bubble, the PSW radially expands from the torus center. A part of the PSW travels radially inwards and focuses on the symmetry axis (Fig. 7A.2). This focus point moves upwards along the symmetry axis, impacts the bubble surface on the south pole, and reflects as a rarefaction wave (Fig. 7A-B.3). By this center impact (CI), the bubble surface strongly accelerates and produces the observed micro-jets (Fig. 7B.1-2). Besides the radially inward focusing of the PSW on the symmetry axis, the PSW first impacts the bubble surface off-axis and reflects as a rarefaction wave (Fig. 7A.1-3). This off-axis impact (OAI) initiates a radial inward contraction of the bubble surface, forming a mushroom-like bubble shape for small standoff distances (Fig. 7B-D.1). Even though we observe an upward jet on the symmetry axis within the bubble, the radial inward contraction prevents stable bubble piercing.

For the investigated standoff distances, γ>1, the maximum bubble size, $R_{b,\max}$ is approximately the same. Since the toroidal bubble is always initiated at the same radial position, ${\overset{ˆ}{r}}_{t,0}$, and with the same initial radius, ${\overset{ˆ}{R}}_{t,0}$, the center impact on the bubble surface always occurs at the same radial coordinate, ${\overset{ˆ}{r}}_{\mathrm{ci}}$, however, the impact time, ${\overset{ˆ}{t}}_{\mathrm{ci}}$, linearly increases with the standoff distance:

(11) $${\overset{ˆ}{r}}_{\mathrm{ci}}=0$$

(12) $${\overset{ˆ}{t}}_{\mathrm{ci}}={\left({\left(\gamma -1\right)}^{2}+{\left({\overset{ˆ}{r}}_{t,0}-{\overset{ˆ}{R}}_{t,0}\right)}^{2}\right)}^{1/2}∼\gamma .$$

For the off-axis impact, the time, ${\overset{ˆ}{t}}_{\mathrm{oai}}$, and radial position, ${\overset{ˆ}{r}}_{\mathrm{oai}}$, solely depend on the initial standoff distance. We can estimate these quantities as

(13) $${\overset{ˆ}{r}}_{\mathrm{oai}}=\frac{{\overset{ˆ}{r}}_{t,0}-{\overset{ˆ}{R}}_{t,0}}{\sqrt{{\left({\overset{ˆ}{r}}_{t,0}-{\overset{ˆ}{R}}_{t,0}\right)}^{2}+\gamma^{2}}}∼\frac{1}{\gamma }$$

(14) $${\overset{ˆ}{t}}_{\mathrm{oai}}={\left(\gamma^{2}+{\left({\overset{ˆ}{r}}_{t,0}-{\overset{ˆ}{R}}_{t,0}\right)}^{2}\right)}^{1/2}-{\overset{ˆ}{R}}_{t,0}-1∼\gamma .$$

Hence, the radial position of the OAI increases inversely proportional with decreasing standoff distances, enhances the squeezing of the lower bubble surface, and prevents a stable micro-jet formation. This effect is further enhanced by flattening the lower bubble side when γ→1. From Eq. (12) and Eq. (14), we find that the impact time linearly increases with the standoff distance. Therefore, the impact strength reduces in both cases approximately with at least 1/γ due to geometric damping of shock waves in the liquid [56]. Hence, the formation of the micro-jet is a balance between shock-wave dampening in the liquid and radial squeezing of the lower bubble surface. First, the reduced dampening of the PSW increases the jet velocity for smaller standoff distances before the enhanced off-axis impact weakens a further jet amplification or even prevents it.

When we follow the focus point on the symmetry axis (Fig. 7A.1-3), we observe that it broadens during its upward motion, forming a stable Mach stem, which has recently also been observed by Liu et al. [33] during the collapse of a toroidal cavitation bubble. No stable Mach stem forms for small standoff distances, and the center impact is more point-like. This will cause the initiation of thinner micro-jets and further reduce jet stability. With increased standoff distance from the wall, the appearance of the Mach stem enhances and the center impact induces broader jets due to its enlarged radial extent (Fig. 7C.1-3).

From correlations Eqs. (11)–(14), we find that the radial position and impact time strongly depend on the initial geometry of the toroidal bubble, particularly its major radius. For smaller major radii, two effects may amplify the jet speed. First, the inward-moving PSW experiences less dampening, increasing pressure at the focus point on the symmetry axis. Second, the radial position of the off-axis impact on the bubble surface decreases, reducing radial squeezing. This may also lead to the formation of thicker jets and improve jet stability. The initial minor radius of the toroidal bubble and, correspondingly, the initial toroidal bubble energy may also influence the jet strength, primarily affecting the initial plasma pressures. Liu et al. [33] studied the influence of the aspect ratio $r_{t,0}/R_{t,0}$ of the toroidal bubble on the collapse pressures and found a strong influence on the peak pressures in the torus center. Since these pressure peaks primarily drive the micro-jet ejection, we expect them to significantly influence it. Furthermore, detailed knowledge of the experiment’s initial toroidal bubble size and shape is needed to improve the calibration of the simulation.

**Fig. 8:**
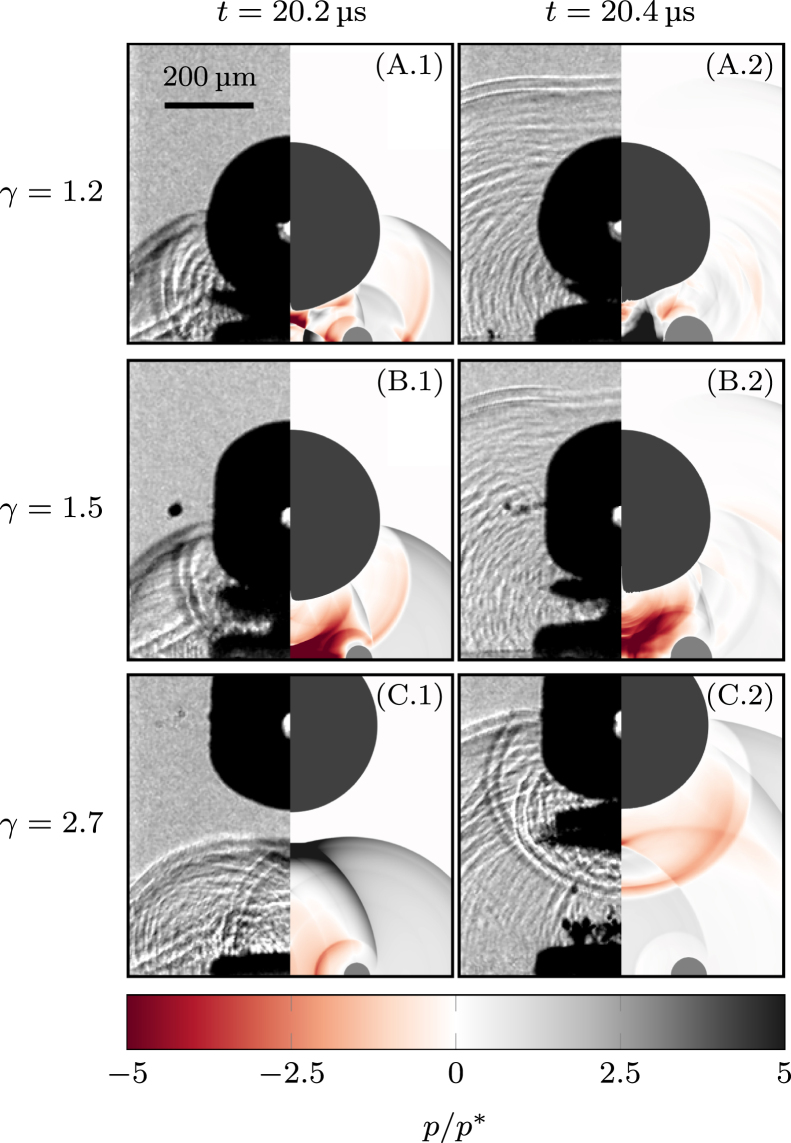
Wave dynamics and formation of secondary cavitation within the first 400ns after toroidal bubble initiation for three different standoff distances, γ≈1.2, γ≈1.5, and γ≈2.7. High-speed side-view dynamics of the experiment (left half of each image) and visualization of the normalized pressure field in the simulation (right half of each image). Time instants and color bar of the pressure field of all images are given at the top and at the bottom, respectively. In (A.1), a scale bar for all images is shown.

In Fig. 6, we observe that simulation and experiment show similar trends for the jet velocity over the nondimensional standoff distance but differ quantitatively. We attribute these differences mainly to two physical phenomena that are suppressed in the simulation due to the choice of numerical model: (hyper)elastic wall deformation and spontaneous secondary cavitation. In Fig. 8, we show the juxtaposition of simulation and experiment within the first 400ns after toroidal bubble initiation.

After the impact of the PSW on the bubble surface, it reflects as a strong rarefaction wave, stretching liquid molecules. These high tensile stresses up to GPa may initiate cavitation nucleation. Since the PSW first impacts the bubble at a certain radial distance from the symmetry axis (Fig. 7), nucleation starts off-axis and moves radially inwards, reaching the peak tensile stresses on the symmetry axis. This leads to curved cavitation clouds following the evolution of the rarefaction wavefront (Fig. 8A-B.1). The radial extent of the cavitation cloud, primarily depends on the tensile strength and, therefore, reduces with increasing radial distance from the symmetry axis. Due to the wall-attached toroidal bubble, a SchHW forms in the liquid. This surface wave further limits the radial extent of the cavitation cloud (experiment in Fig. 8A-C.1). Since the highest tensile stresses are found on the symmetry axis, secondary cavitation nucleation dominates on the axis and even extends into the center of the toroidal bubble Fig. 8(B.1-2).

The formation of secondary cavitation may have a significant effect on the initiation of the observed micro-jets. Due to the time differences between off-axis impact and center impact on the axis of the PSW, waves may be trapped between bubble and secondary cavitation cloud. Thus, the waves undergo multiple reflections between bubble surface and cavitation cloud, amplifying the micro-jet formation. This would move the appearance of the micro-jets to larger standoff distances and amplify the jet velocity [70]. Since the radial position of the PSW moves radially inward for larger standoff distances, the jet width is also influenced by this amplification effect, leading to thicker jets at larger standoff distances, as observed in the experiments. However, the off-axis start of the nucleation also enhances the radial squeezing of the lower bubble surface by multiple wave reflections, moving the rapid decrease of the jet velocity below the peak velocities to larger standoff distances. Another amplification factor may be the SchHW, which impacts and reflects from the bubble surface shortly after the cavitation cloud has formed Fig. 8(A-C.1). Hence, these surface waves further accelerate the lower part of the bubble and increase jet velocity.

We find that peak jet velocities occur within a non-dimensional standoff distance range of γ≈1.4–1.5, which aligns with the peak velocities observed for an anti-phase spherical bubble pair in a free field [38], [43], [44]. Fan et al. [44] report peak velocities up to 1000m/s at $\gamma_{\mathrm{ff}}=\gamma /2\approx 0.75$, where a micro-jet forms opposite to the growth direction of the second bubble. The jet is initiated by the neck collapse of the second bubble due to a perfectly timed CSW from the pierced first spherical bubble. Slightly lower velocities are reported by Han et al. [38] and Mishra et al. [43] for larger bubbles. On the contrary, in this paper, jets are initiated by the torus shock wave impacting the bubble surface, forming a micro-jet in the toroidal growth direction. Compared to the anti-phase bubble pair scenario, these micro-jets are less stable, thinner, and shorter, attributed to an impact-driven initiation mechanism rather than continuous feeding after neck collapse seen in the anti-phase scenario. The appearance of these supersonic micro-jets is limited to a narrow standoff distance range of $\gamma_{\mathrm{ff}}\approx 0.7\text{–}0.8$, diminishing for smaller and larger distances. The presence of this very small range corresponds well to our observations.

After the micro-jet is formed, a subsequent broad jet pierces the bubble, driven by the expansion of the wall-bounded toroidal bubble along the symmetry axis. Similar jetting effects are observed in single cavitation bubbles collapsing near a solid wall. These broad jets form at sufficient distances from the wall due to the pressure difference across the bubble surface, with higher pressures above the bubble [12], [16], [19]. For bubbles close to the wall (γ≈0.3), jet velocities reach around 30m/s [12], [16], increasing to O(100)m/s at larger standoff distances (γ>1) [19]. In this study, jet initiation is driven by the expansion of the toroidal bubble. At larger standoff distances, similar jet velocities ($u_{\mathrm{jet}}\approx \text{100}\;\text{m/s}$) are observed, as the bubble experiences a similar far-field driving force. However, decreasing the distance from the wall increases the strength of toroidal bubble expansion experienced by the spherical bubble, enhancing the broad jet velocity up to $u_{\mathrm{jet}}\approx \text{300}\;\text{m/s}$. This closely relates the broad jet formation to shock-induced bubble collapse [22], [23]. There, a high post-shock velocity drives the formation of broad liquid jets pierces the gas bubble. The jet velocity reaches values around O(1000)m/s [22], [23], [71] and decreases with smaller shock strengths [22]. Similar trends are found here, where smaller standoff distances increase the pushing effect from the wall-bounded toroidal bubble and, thus, enhance jet velocity. Compared to the shock-induced scenario of millimeter-sized bubbles, smaller absolute jet velocities are found [22] because the driving force reduces over time, whereas the post-shock velocity continuously feeds the jet. However, the jet velocity in the shock-induced case also strongly depends on the bubble size, where higher jet velocities are found for larger bubbles under the same shock strength [20].

Compared to single cavitation bubble dynamics, the interaction of multiple bubbles increases the parameter space significantly. However, we have found that the micro-jets are most dominant in the anti-phase scenario, where the toroidal bubble is initiated right when the bubble reaches its maximum. We attribute that to the non-moving bubble interface for these delay times, enhancing the momentum exchange from the impacting shock waves on the bubble surface. Spherical bubble expansion is not yet complete for shorter delay times, and jet formation is reduced due to a counter-moving interface. Conversely, the spherical bubble already collapses for larger delay times, and the surface shrinks away from the wall, decreasing the relative velocity between impacting waves and the bubble surface. This observation corresponds to other bubble pair arrangements [38], [43], [44].

### 4.3 Spatio-temporal bubble dynamics

**Fig. 9:**
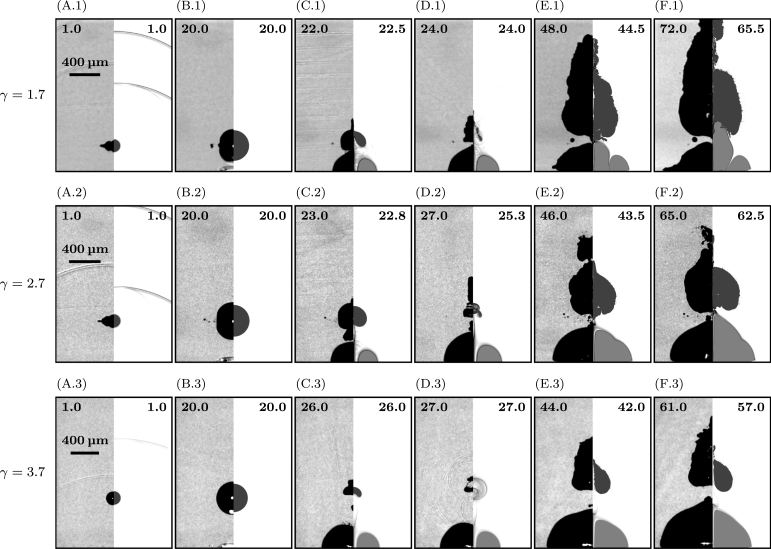
Bubble dynamics until second expansion maximum of the spherical bubble for three nondimensional standoff distances, γ≈1.7 (A-F.1), γ≈2.7 (A-F.2), and γ≈3.7 (A-F.3): High-speed side-view shows the experiment (left half of each image) and visualization of numerical Schlieren in the simulation (right half of each image). The experimental shadowgraphs depict a bubble projection, while the simulation a plane cutting the bubble. Time instants are given in microseconds in the upper of each image, and in (A.1-3) a scale bar for every standoff distance is shown.

**Fig. 10:**
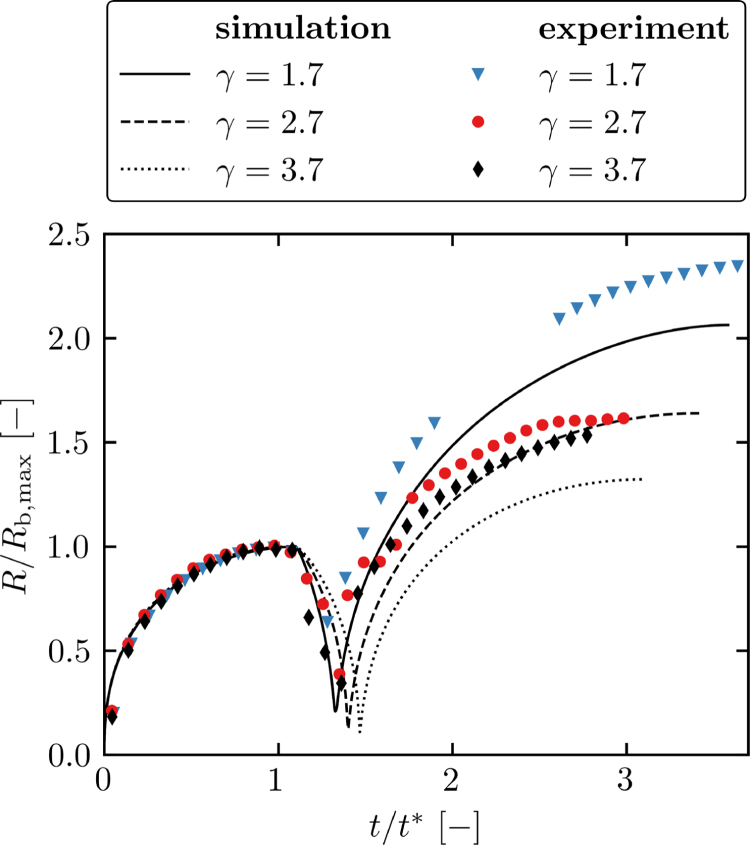
Normalized equivalent radius of the spherical bubble, $R_{b}/R_{b,\max}$, as function of normalized time, $t/t^{*}$. The evolutions are shown for three standoff distances, γ≈1.7, γ≈2.7, and γ≈3.7. Note that in the simulations, the equivalent radius is computed from the bubble volume, whereas the experiment uses an ellipsoidal fit.

After the formation of the initial micro-jet and the subsequent broad jet, the remaining pierced spherical bubble collapses, initiates bubble rebound, and reaches sizes greater than the original size after the first expansion. In Fig. 9, the dynamics of the toroidal–spherical interaction scenario are visualized starting from the initiation of the spherical bubble until it reaches its maximum rebound size. Additionally, we show in Fig. 10 the evolution of the normalized equivalent radius, $R_{b}/R_{b,\max}$, over the normalized time, $t/t^{*}$. The numerical equivalent radius is computed through the bubble volume as $R_{b,\mathrm{num}}={\left(3/\left(4\pi \right)V_{b}\right)}^{1/3}$. In the experiment, the equivalent radius is approximated by ellipsoidal fitting, $R_{b,\exp}={\left(a_{b}b_{b}^{2}\right)}^{1/3}$, where $a_{b}$ is the major and $b_{b}$ is the minor semi-axis of the fitting ellipsoid.

From Fig. 9, Fig. 10, we find that the evolution of the spherical-bubble radii can be split into three stages. First, initial undisturbed bubble expansion until the first maximum. Second, initiation of the toroidal bubble and formation of micro-jets and subsequent broad jets, resulting in an accelerated collapse of the spherical bubble. Third, after spherical bubble collapse, the bubble undergoes an amplified expansion.

Simulation and experiment agree reasonably well, representing similar trends for the three stages where quantitative differences are visible. As previously shown, the micro-jet formation is observed in the simulation in a much smaller range of standoff distances, and thinner jets are present. This also has a consequence for the amplified expansion behavior. When the expansion starts (Fig. 9D.1-3), we can divide the bubble into two parts: a lower central part represented by the toroidal bubble and an upper part resulting from the micro-jet. In the following expansion phase (Fig. 9E.1-3), both parts grow in size. The simulation accurately captures the expansion of the central lower part. However, the upper part is significantly thinner or absent (Fig. 9F.2-3), which is caused by non-existing micro-jets for larger standoff distances. For smaller standoff distances, better agreement is observed (Fig. 9F.1).

The strong amplification of the bubble after rebound contrasts with single bubble dynamics near a solid wall, where the rebound size remains finite and smaller than the original maximum expansion size after jet piercing due to acoustic radiation of strong collapse shock waves [12], [16], [72]. In single-cavitation bubble dynamics, the post-collapse evolution primarily depends on the standoff distance, distinguishing free-vortex (γ≲1.3) and wall-vortex (γ≳1.35) scenarios [27], [28]. In the wall-vortex case, the bubble remains attached to the wall after rebound, with its size confined by the wall. Even in the free-vortex scenario, the bubble size remains finite due to the absence of an external driving force to amplify the rebound. Wang [72] show that for decreasing standoff distances, acoustic radiation reduces carrying more energy within the liquid jet that pierces the bubble. Thus, the bubble rebounds to larger sizes compared to a non-jetting bubble, but still remains smaller than the original size of maximum expansion.

In the toroidal–spherical bubble interaction, the micro-jet leads to an elongation of the spherical cavitation bubble. Afterwards, the subsequent broad jet and the expansion of the toroidal bubble continuously push liquid upward. This push significantly stretches the cavitation bubble in wall-normal direction during the expansion in the rebound phase. At the same time, radial growth of the spherical bubble is initiated after its collapse. However, for smaller standoff distances the faster jets and the push of toroidal bubble carry more energy. Thus, the losses through acoustic radiation during the collapse are effectively reduced allowing larger expansions for smaller standoff distances [72]. Additionally, the proximity and expansion of the toroidal bubble generates a complex pressure field around the spherical bubble which may support size amplification.

This contrasts with the anti-phase scenario of two cavitation bubbles in the free field, where no amplification of the first bubble is observed [38], [44]. The main difference lies in the formation of the micro-jet, which is counter-directed to the growth of the second bubble. Hence, less liquid is pushed along the symmetry axis after jet formation through the pierced first bubble, reducing its stretching in axial direction and size amplification.

Bubble elongation in jet direction has previously been observed for shock-induced bubble collapses [20], [21], [26]. When the shock impacts the bubble in the free field, thin elongated jets form in shock direction, but no amplification of the bubble volume is found [20], [21]. Koukas et al. [26] show that shock-induced collapse near a soft tissue may lead to bubble elongation within the tissue material, forming multiple toroidal bubbles. However, no volumetric amplification is visible compared to the initial bubble size.

During the three stages of spherical bubble dynamics, the wall-bounded toroidal bubble undergoes a single expansion phase. We find that the simulation and experiment show similar expansion behaviors. In the late-stage dynamics, the toroidal bubble in the simulations continues to grow in height. In contrast, the height is bounded in the experiments (Fig. 9F.1). This observation has already been found during calibration (Appendix B), where a cusp forms at the symmetry axis. We attribute this fact to the imposed axisymmetric setup, where a small liquid column remains at the torus center, preventing the toroidal bubble from merging into a simply connected bubble. However, when we compare the width of the toroidal bubble, we find much better agreement due to the unbounded expansion in radial direction.

The above observations directly transfer to the evolution of the equivalent radius of the bubble (Fig. 10), where the experiment shows larger amplification rates than the simulation. When we compare the trends in the amplification rate and expansion time, we find that the simulation reveals an approximately linear increase in the amplification rate with the standoff distance. In the experiment, this trend is less pronounced, indicating the complexity of the expansion process after jet formation and its dependency on various parameters of the experimental setup. In contrast, the simulation always employs identical initial conditions for different standoff distances. However, the main characteristics agree, where larger amplification rates and longer expansion times are reached for smaller standoff distances. Also, the linear correlation of the expansion time with the bubble size accurately matches. This behavior also applies to unbounded expansion of a spherical bubble, where the collapse and expansion time linearly increases with the maximum bubble radius.

**Fig. 11:**
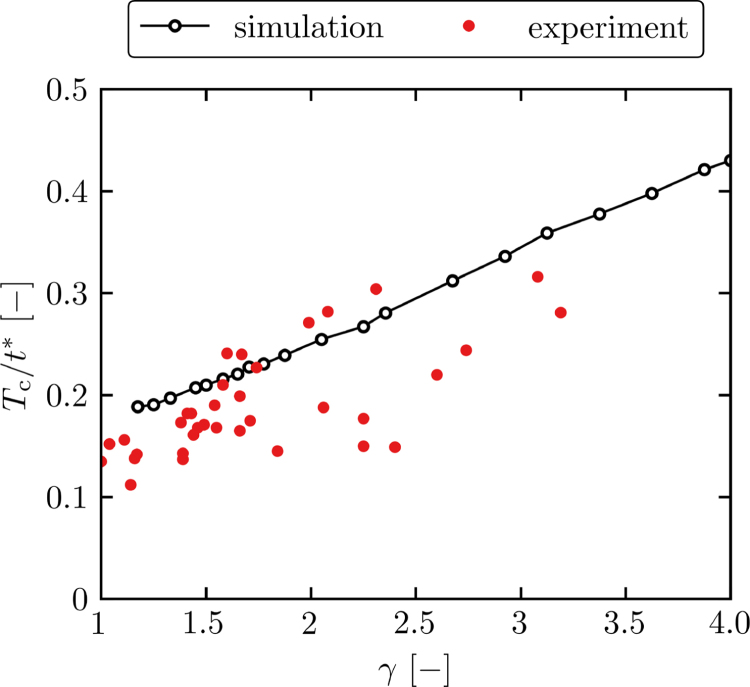
Collapse-time shortening, $T_{c}/t^{*}$, over the nondimensional standoff distance for the simulation and the experiment. The collapse time of the spherical bubble is deduced from the point, where CSWs are visible for the first time.

In the second stage of the evolution cycle, we find an accelerated collapse of the spherical bubble. In Fig. 11, we plot the collapse-time shortening, $T_{c}/t^{*}$, over the nondimensional standoff distance, where the collapse time $T_{c}$ represents the elapsed time from toroidal bubble initiation to spherical bubble collapse estimated by the presence of CSWs (e.g., Fig. 4g). The acceleration of the spherical bubble collapse is mainly influenced by the formation of the broad jets. After jet piercing, the remainder of the spherical bubble further collapses due to the upward liquid motion induced by the jet and continuously fed from the expansion of the wall-bounded toroidal bubble.

The collapse-time shortening linearly decreases with smaller standoff distances in both the simulation and experiment. This contrasts to the trends of the subsequent broad jet velocity, which non-linearly increases with lower standoff distances (Fig. 6). However, we also find more complex wave dynamics below the bubble for smaller standoff distances due to the proximity to the wall. Hence, the formation process of the jet, which is driven by a high-pressure region below the bubble, decelerates for smaller standoff distances, balancing the faster final jet velocities. We find that the collapse-time shortening is greater in the experiments, i.e. the spherical bubble collapses earlier, which we attribute to mainly two effects. First, the micro-jet velocity is faster in the experiments initiating bubble collapse earlier. Second, the wall-normal expansion rate of the toroidal bubble is smaller in the simulations (see Appendix B) reducing the velocity of the liquid that is continuously pushed through the bubble.

### 4.4 Discussion on experimental and numerical comparison

Comparing simulation results with experimental observations, we find good agreement for the overall dynamics (Fig. 9). The simulation accurately captures initial wave dynamics after the toroidal bubble initiation, jet formation, spherical bubble collapse, and amplification during rebound. Hence, these phenomena are predominantly driven by the shock wave impact and expansion from the subsequently generated toroidal bubble. However, we find quantitative differences (e.g., Fig. 6, Fig. 11) indicating the relevance of various physical effects.

In the experiments, fundamental dynamics remain consistent across variations, though quantitative differences are observed, particularly in long-term dynamics and bubble amplification rates. A crucial parameter influencing bubble dynamics is the toroidal bubble expansion rate, which depends on beam shaping accuracy, focal point precision, and energy distribution within the ring-shaped plasma. Variations in the bubble’s size at its first maximum also complicate direct comparisons with simulations for similar standoff distances (Fig. 9B.1-3). Additionally, achieving the employed axisymmetric setup experimentally is challenging. Hence, the simulation creates an idealized scenario for the anti-phase toroidal–spherical interaction scenario.

Furthermore, secondary cavitation significantly influences the formation of micro- and broad jets. Because jet timing and velocity, essential for the initiated collapse of the pierced spherical bubble and subsequent bubble dynamics, rely on secondary cavitation, accurately modeling its inception in simulations is essential [73]. However, such modeling is constrained by limited knowledge of bubble nucleation, which is difficult to estimate reliably for the liquid under investigation.

To better understand the differences between numerical and experimental results, particularly the occurrence of micro-jets, it would be valuable to vary the initial toroidal energy or equivalently, the toroidal bubble size. However, with the current experimental setup, such variation is complex, as beam shaping, laser pulse duration, and liquid breakdown thresholds must be carefully matched to realize the changes. This adjustment of initial conditions is more straightforward in the simulations once a suitable calibration for a non-interacting toroidal bubble is achieved. As shown in Appendix B, matching toroidal bubble dynamics to experiments can be effectively managed by finding a suitable initial bubble size for a fixed initial pressure that recovers the outer radius and collapse time of the toroidal bubble. However, discrepancies in wall-normal expansion rates remain leading to deviations in the height of the toroidal bubble. Accurate calibration requires consideration of additional factors, including complex equations of state for water and vapor [74], vapor–gas mixtures [56], [75], phase-change effects at bubble interfaces [12], [54], [55], [57], laser effects or equivalently, non-uniform initial pressure/energy distributions [76], the bubble’s initial wall distance, and shock-wave reflections from the walls of the experimental cuvette [77]. These adjustments require precise experimental data, such as the initial plasma shape and position [78], [79], detailed knowledge about the bubble content, and exact evaporation/condensation rates throughout the bubble evolution [80], [81]. However, such data are difficult to obtain in experiments and are rarely available in the literature. Consequently, a consistent study allowing a mapping to this scenario does not yet exist, leaving exact calibration as an open question for future research.

## 5 Conclusion

In this work, we investigate the anti-phase interaction of a wall-bounded toroidal bubble with a previously expanding spherical bubble near a solid wall by varying the initial standoff distance of the spherical bubble. Micro-jets form on the upper bubble side shortly after toroidal bubble initiation, with jet velocities reaching up to 900m/s. The ejection of these jets is driven by the focus of the torus plasma shock wave on the symmetry axis, impacting the lower bubble side. The peak jet velocities occur at standoff distances around γ≈1.4–1.5, where shock wave dampening balances radial squeezing of the lower bubble side, resulting in reduced jet speeds for smaller and larger standoff distances. Additionally, the micro-jet broadens at larger standoff distances due to forming a broader Mach stem from the converging plasma shock wave on the symmetry axis. After the micro-jet pierces the spherical bubble, a subsequent broad jet emerges due to the expansion of the wall-bounded toroidal bubble, which ultimately initiates the collapse of the pierced spherical bubble. Following the bubble collapse, we observe significant amplification rates of the spherical bubble, leading to bubble sizes two to three times larger than the original maximum size. This work opens a new potential pathway for controlled micro-jet formation and bubble size amplification. It also provides some first experimental insights of how well-controlled experiments on energy focusing from torus shock wave interacting with a spherical bubble could be achieved. The experiments reveal that secondary cavitation may contribute to the resulting dynamics. Similar effects may occur in shock driven toroidal–spherical bubble interaction, which have been proposed for inertial confined fusion [82].

## CRediT authorship contribution statement

**Jaka Mur:** Writing – review & editing, Writing – original draft, Visualization, Validation, Investigation, Data curation, Conceptualization. **Alexander Bußmann:** Writing – review & editing, Writing – original draft, Visualization, Software, Methodology, Investigation, Formal analysis, Data curation, Conceptualization. **Thomas Paula:** Writing – review & editing, Validation, Software. **Stefan Adami:** Supervision, Writing - Review & Editing. **Nikolaus A. Adams:** Writing – review & editing, Supervision, Resources, Funding acquisition. **Rok Petkovsek:** Writing – review & editing, Validation, Supervision, Resources, Project administration, Methodology, Funding acquisition, Formal analysis, Conceptualization. **Claus-Dieter Ohl:** Writing – review & editing, Supervision, Resources, Funding acquisition.

## Declaration of competing interest

The authors declare that they have no known competing financial interests or personal relationships that could have appeared to influence the work reported in this paper.

## Figures and Tables

**Fig. A.12:**
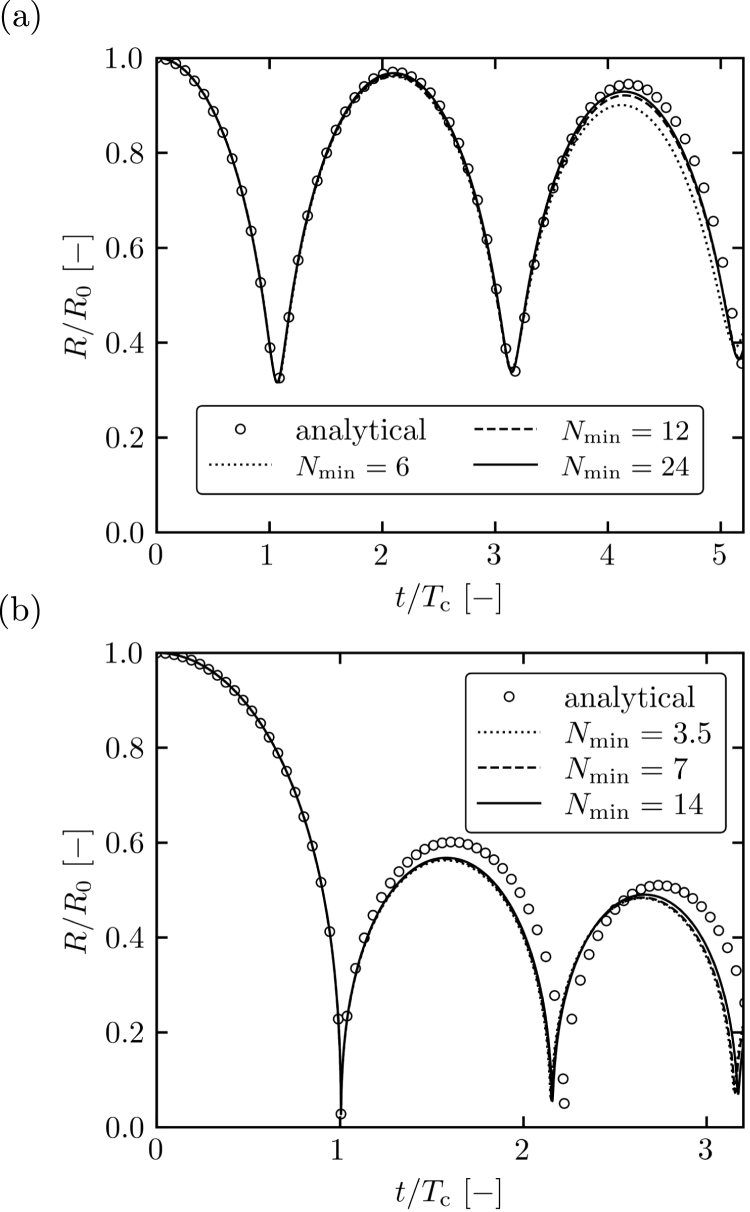
Convergence study of different mesh resolutions and comparison to analytical solution for a weak collapse scenario (a) and strong collapse scenario (b). The weak scenario is defined by a initial pressure jump across the bubble surface of $p_{*}=p_{\infty }/p_{b}\approx 10$ and in the strong scenario we enforce a jump of $p_{*}\approx 1000$, where $p_{\infty }$ is the external liquid pressure and $p_{b}$ the internal bubble pressure. Shown are the normalized temporal evolution, $t/T_{c}$, over the normalized equivalent radius, $R_{b}\left(t\right)/R_{b,0}$, where $R_{b,0}$ is the initial bubble radius and $T_{c}=0.915R_{b,0}\sqrt{\rho_{\infty }/\left(1-p^{*}\right)}$ is the Rayleigh collapse time with $\rho_{\infty }$ being the liquid density [47]. For the analytical solution, we employ the Gilmore model with identical parameters as used in the simulation [74].

**Fig. B.13:**
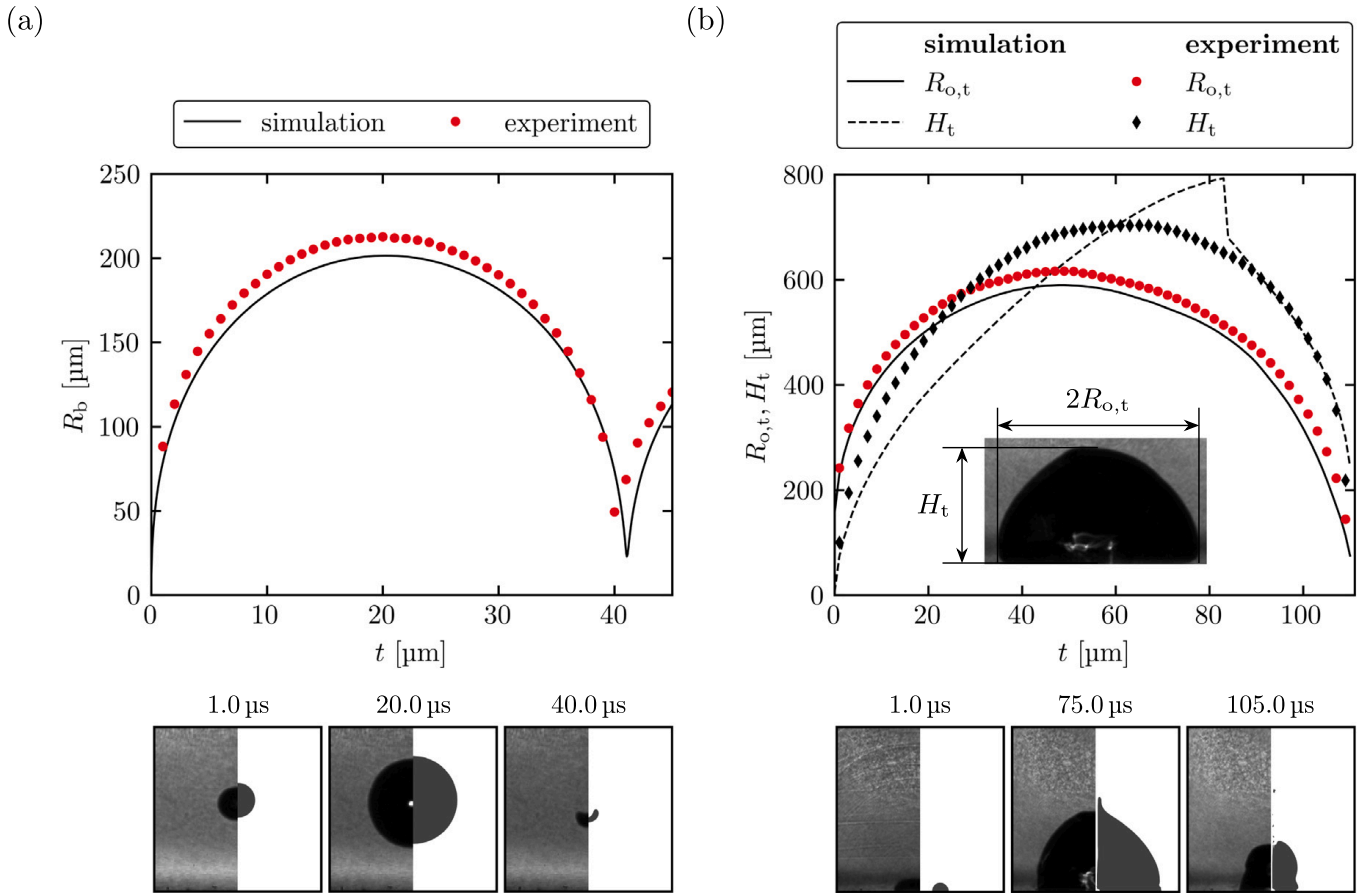
Comparison between simulation and experiment for the dynamics of a single laser-induced cavitation bubble in water: (a) Temporal evolution of the equivalent radius, $R_{b}\left(t\right)$ of the spherical cavitation bubble, initiated at $\gamma =D_{0}/R_{b,\max}\approx 2$ above the wall. (b) Temporal evolution of outer radius, $R_{o,t}$, and height, $H_{t}$, of the toroidal cavitation bubble initiated at the wall. At the bottom of each plot, the juxtaposition of experiment (left half of each image) and simulation (right half of each image) is visualized at three time instants.

**Fig. C.14:**
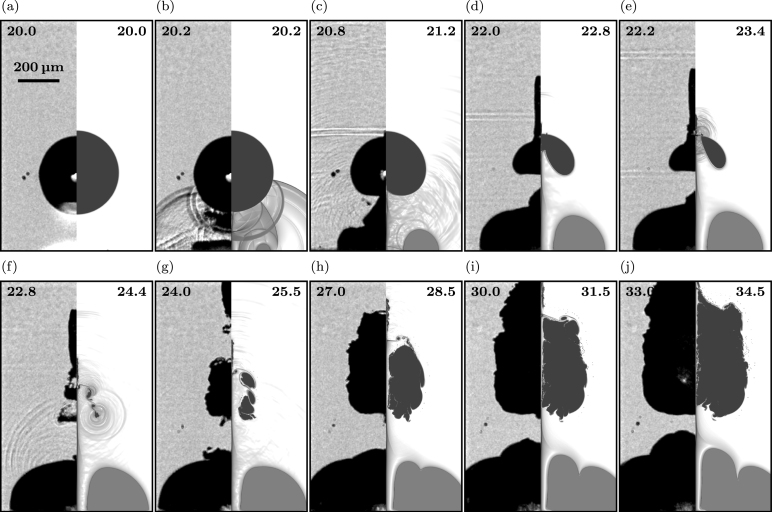
Early stage dynamics comparing simulation and experiment of the anti-phase toroidal–spherical cavitation bubble scenario for a nondimensional standoff distance of γ≈1.85: High-speed side-view dynamics of the experiment (left half of each image) and visualization of numerical Schlieren in the simulation (right half of each image). The experimental shadowgraphs show a bubble projection, while the simulation shows a plane cutting the bubble. Time instances are given in micro seconds at the top of each image, and in (a) a scale bar for all images is shown.

**Fig. C.15:**
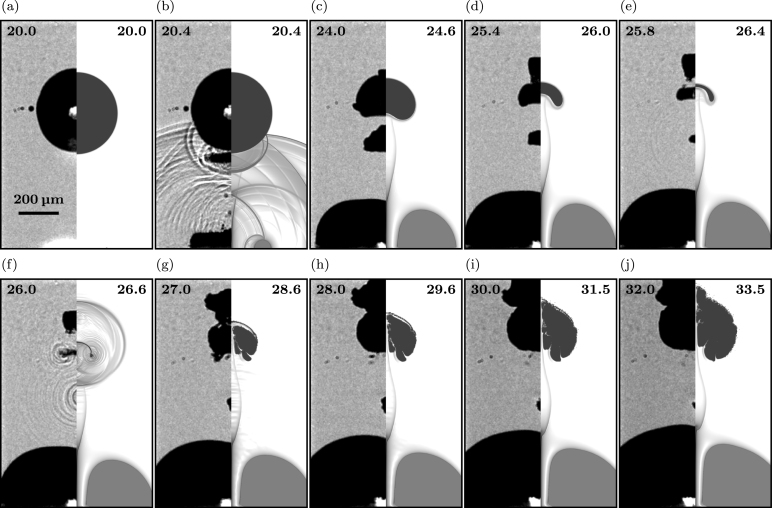
Early stage dynamics comparing simulation and experiment of the anti-phase toroidal–spherical cavitation bubble scenario for a nondimensional standoff distance of γ≈3.2: High-speed side-view dynamics of the experiment (left half of each image) and visualization of numerical Schlieren in the simulation (right half of each image). The experimental shadowgraphs show a bubble projection, while the simulation shows a plane cutting the bubble. Time instances are given in micro seconds at the top of each image, and in (a) a scale bar for all images is shown.

## Appendix A Resolution study based on Rayleigh collapse scenario

We investigate the effect of the minimal cell size $\Delta x_{\min}$ in the simulation with a weak and strong Rayleigh collapse scenario. In the weak scenario, an initial spherical bubble with $R_{b,0}=\text{250}\;\text{µm}$ collapses due to a pressure jump across the bubble interface of $\overset{ˆ}{p}=p_{\infty }/p_{b}\approx 10$, where $p_{\infty }$ is the external liquid pressure and $p_{b}$ the internal bubble pressure. In the strong scenario the initial radius is set to $R_{b,0}=\text{350}\;\text{µm}$ and the pressure jump is $\overset{ˆ}{p}\approx 1000$.

Fig. A.12 shows the normalized temporal evolution of the normalized equivalent radius $R_{b}\left(t\right)={\left(3/\left(4\pi \right)V_{b}\left(t\right)\right)}^{1/3}$, where $V_{b}\left(t\right)$ is the bubble volume, for different resolutions. We characterize the resolutions by the resolution of the minimal radius, $N_{\min}=R_{b,\min}/\Delta x_{\min}$. In the strong collapse scenario we observe that only a few cells are required to reach convergence. Similarly, the first rebound is accurately captured with the lowest resolution in the weak collapse scenario. For more oscillation cycles, a slightly higher resolution is needed. Hence, we conclude that a minimal resolution of $N_{\min}>12$ is sufficient to capture the main cavitation bubble dynamics.

Comparing the simulation results to the analytical Gilmore model with identical parameters as used in the simulation [74], we find that the simulation for both scenarios accurately predicts the first collapse. In the rebound phases, deviations are visible in the bubble radius and cycle time, which increase for larger initial pressure jumps and longer simulation times. However, these deviations are expected since the outward propagation of pressure waves is only captured up to second-order by the Gilmore model leading to larger rebound radii and corresponding longer cycle times [55], [83].

## Appendix B Calibration of initial conditions for single spherical and toroidal cavitation bubbles

We have calibrated the spherical and toroidal bubbles individually to find suitable initial conditions for the simulation. For the spherical bubble, a single bubble evolution has been used with a non-dimensional standoff distance of γ≈2 as it represents a good reference scenario for the investigated standoff distances in the range γ∈[1,4]. The single toroidal bubble dynamics are initiated at the wall, as done in the parametric study. The initial sizes are $R_{b,0}=\text{8.25}\;\text{µm}$ for the spherical bubble and minor and major radius of $r_{t,0}=\text{5.1}\;\text{µm}$ and $R_{t,0}=\text{150}\;\text{µm}$ for the toroidal bubble.

In Fig. B.13, the geometrical evolution of both the spherical and toroidal bubble is shown. At the bottom of every plot, we show the juxtaposition of experiment and simulation in three instances. We observe that for the spherical bubble, the calibration yields excellent results for the bubble size and oscillation period. The bubble size in the simulation is slightly below the experimental size with an absolute error of approximately 5%. The oscillation period also gives reasonable agreement with an approximate error of 2.5%.

Similar trends are also observed for the outer radius of the toroidal bubble, where the simulation captures the oscillation period and size accurately. Again, the simulation has slightly smaller dimensions. Contrary to the radius, the height shows apparent differences compared to the experiment. During the initial expansion phase, the height of the simulated bubble shows a smaller growth rate. We account that to the complex process of plasma initiation in the experiments, where, e.g., a shock wave is emitted into the wall and reflects from it, potentially enhancing the wall-normal growth.

We also observe differences in the maximum height of the cavitation bubble, where we find that a cusp forms in the simulation (snapshot at t=75µs in Fig. B.13(b)). We account that to the axisymmetric setup in the simulation, where the experiment always introduces asymmetries preventing the formation of a stable liquid column in the toroidal bubble center. However, during the collapse of the toroidal bubble, the cusp separates from the main bubble, leading to an accurate predicted height in the final stages of the bubble evolution. The formation of the cusp also leads to a slightly different collapse behavior of the toroidal bubble, where in the experiment the toroidal bubble collapses from the top and in the simulation circumferential pinching is observed.

## Appendix C Complementary early stage dynamics

In Section 4.1, we analyze the dynamics of the toroidal–spherical bubble interaction scenario for three distance nondimensional standoff distances, γ≈1.2, γ≈1.5, and γ≈2.7. As a complementary study, we present here two additional standoff distances γ≈1.85 (Fig. C.14) and γ≈3.2 (Fig. C.15).

The dynamics immediately following the toroidal bubble initiation are governed by the complex wave interactions resulting from the plasma shock wave and its subsequent reflections on the bubble and toroidal bubble surfaces (Fig. C.14a-c and Fig. C.15a-b). We observe the formation of a secondary cavitation cloud between the toroidal and the spherical bubble, with the cloud being larger and evolving independently of the spherical bubble at larger standoff distances (Fig. C.14f).

In the experiment, for both standoff distances, an initial micro-jet appears on the upper side of the bubble. This jet is longer and thinner for the smaller standoff distance (compare Fig. C.14c-e and Fig. C.15c-e). In the simulation, a micro-jet only appears for γ≈1.85, forming later and being significantly smaller than in the experiment. During the formation of the micro-jet, a subsequent broad jet develops, pierces the bubble on the upper side, and separates the micro-jet from the bubble (compare Fig. C.14f and Fig. C.15e). Following the piercing of the broad jet, the remaining toroidal bubble collapses, rebounds, and amplifies in size (compare Fig. C.14g-j and Fig. C.15g-j). Depending on the proximity of the micro-jet to the main bubble, merging of jet and bubble may occur (Fig. C.15g). The amplification of the rebound phase mainly depends on the wall distance, with higher amplification rates observed for bubbles closer to the wall.

For the wall-bounded toroidal bubble, the early-stage dynamics follow similar trends. Initially, the toroidal bubble grows independently in width and height. The formation of a constriction and the subsequent generation of a cusp in the toroidal bubble center depend on the standoff distance; no cusp is visible at large standoff distances (compare Fig. C.14j and Fig. C.15j).

## Appendix D Supplementary data

Video S1 Numerical bubble dynamics (3D) for γ≈1.2. The cut planes show numerical Schlieren (left) and velocity magnitude (right).

Video S2 Numerical bubble dynamics (3D) for γ≈1.5. The cut planes show numerical Schlieren (left) and velocity magnitude (right).

Video S3 Numerical bubble dynamics (3D) for γ≈1.85. The cut planes show numerical Schlieren (left) and velocity magnitude (right).

Video S4 Numerical bubble dynamics (3D) for γ≈2.7. The cut planes show numerical Schlieren (left) and velocity magnitude (right).

Video S5 Numerical bubble dynamics (3D) for γ≈3.2. The cut planes show numerical Schlieren (left) and velocity magnitude (right).

Video S6 Experimental early-stage bubble dynamics for γ≈1.2. The cut plane shows high-speed shadowgraphs.

Video S7 Numerical early-stage bubble dynamics for γ≈1.2. The cut plane shows numerical Schlieren.

Video S8 Experimental early-stage bubble dynamics for γ≈1.5. The cut plane shows high-speed shadowgraphs.

Video S9 Numerical early-stage bubble dynamics for γ≈1.5. The cut plane shows numerical Schlieren.

Video S10 Experimental early-stage bubble dynamics for γ≈1.85. The cut plane shows high-speed shadowgraphs.

Video S11 Numerical early-stage bubble dynamics for γ≈1.85. The cut plane shows numerical Schlieren.

Video S12 Experimental early-stage bubble dynamics for γ≈2.7. The cut plane shows high-speed shadowgraphs.

Video S13 Numerical early-stage bubble dynamics for γ≈2.7. The cut plane shows numerical Schlieren.

Video S14 Experimental early-stage bubble dynamics for γ≈3.2. The cut plane shows high-speed shadowgraphs.

Video S15 Numerical early-stage bubble dynamics for γ≈3.2. The cut plane shows numerical Schlieren.

Video S16 Experimental long-time bubble dynamics for γ≈1.7. The cut plane shows high-speed shadowgraphs.

Video S17 Numerical long-time bubble dynamics for γ≈1.7. The cut plane shows numerical Schlieren.

Video S18 Experimental long-time bubble dynamics for γ≈2.7. The cut plane shows high-speed shadowgraphs.

Video S19 Numerical long-time bubble dynamics for γ≈2.7. The cut plane shows numerical Schlieren.

Video S20 Experimental long-time bubble dynamics for γ≈3.7. The cut plane shows high-speed shadowgraphs.

Video S21 Numerical long-time bubble dynamics for γ≈3.7. The cut plane shows numerical Schlieren.

Video S22 Early flow field dynamics for γ≈1.15. The cut planes show pressure (left) and velocity magnitude/arrows (right).

Video S23 Early flow field dynamics for γ≈1.55. The cut planes show pressure (left) and velocity magnitude/arrows (right).

Video S24 Early flow field dynamics for γ≈2.05. The cut planes show pressure (left) and velocity magnitude/arrows (right).

## References

[1] Reuter F., Deiter C., Ohl C.-D. (2022). Cavitation erosion by shockwave self-focusing of a single bubble. Ultrason. Sonochemistry.

[2] Schoppink J., Rivas D.F. (2022). Jet injectors: Perspectives for small volume delivery with lasers. Adv. Drug Deliv. Rev..

[3] Robles V., Gutierrez-Herrera E., Devia-Cruz L., Banks D., Camacho-Lopez S., Aguilar G. (2020). Soft material perforation via double-bubble laser-induced cavitation microjets. Phys. Fluids.

[4] Benjamin T.B., Ellis A.T. (1966). The collapse of cavitation bubbles and the pressure thereby produced against solid boundaries. Phil Truns R. Soc. Lond..

[5] Vogel A., Lauterborn W., Timm R. (1989). Optical and acoustic investigations of the dynamics of laser-produced cavitation bubbles near a solid boundary. J. Fluid Mech..

[6] Brujan E.-A., Nahen K., Schmidt P., Vogel A. (2001). Dynamics of laser-induced cavitation bubbles near an elastic boundary. J. Fluid Mech..

[7] Brujan E.A., Keen G.S., Vogel A., Blake J.R. (2002). The final stage of the collapse of a cavitation bubble close to a rigid boundary. Phys. Fluids.

[8] Tomita Y., Robinson P.B., Tong R.P., Blake J.R. (2002). Growth and collapse of cavitation bubbles near a curved rigid boundary. J. Fluid Mech..

[9] Kröninger D., Köhler K., Kurz T., Lauterborn W. (2009). Particle tracking velocimetry of the flow field around a collapsing cavitation bubble. Exp. Fluids.

[10] Lauterborn W., Lechner C., Koch M., Mettin R. (2018). Bubble models and real bubbles: Rayleigh and energy-deposit cases in a tait-compressible liquid. IMA J. Appl. Math..

[11] Lechner C., Lauterborn W., Koch M., Mettin R. (2019). Fast, thin jets from bubbles expanding and collapsing in extreme vicinity to a solid boundary: A numerical study. Phys. Rev. Fluids.

[12] Lechner C., Lauterborn W., Koch M., Mettin R. (2020). Jet formation from bubbles near a solid boundary in a compressible liquid: Numerical study of distance dependence. Phys. Rev. Fluids.

[13] Sagar H.J., el Moctar O. (2020). Dynamics of a cavitation bubble near a solid surface and the induced damage. J. Fluids Struct..

[14] Trummler T., Bryngelson S.H., Schmidmayer K., Schmidt S.J., Colonius T., Adams N.A. (2020). Near-surface dynamics of a gas bubble collapsing above a crevice. J. Fluid Mech..

[15] Reuter F., Ohl C.-D. (2021). Supersonic needle-jet generation with single cavitation bubbles. Appl. Phys. Lett..

[16] Bußmann A., Riahi F., Gökce B., Adami S., Barcikowski S., Adams N.A. (2023). Investigation of cavitation bubble dynamics near a solid wall by high-resolution numerical simulation. Phys. Fluids.

[17] Sieber A., Preso D., Farhat M. (2023). Cavitation bubble dynamics and microjet atomization near tissue-mimicking materials. Phys. Fluids.

[18] Shi H., Zhang H., Geng L., Qu S., Wang X., Nikrityuk P.A. (2024). Dynamic behaviors of cavitation bubbles near biomimetic surfaces: A numerical study. Ocean Eng..

[19] Supponen O., Obreschkow D., Tinguely M., Kobel P., Dorsaz N., Farhat M. (2016). Scaling laws for jets of single cavitation bubbles. J. Fluid Mech..

[20] Ohl C.D., Ikink R. (2003). Shock-wave-induced jetting of micron-size bubbles. Phys. Rev. Lett..

[21] Sankin G.N., Simmons W.N., Zhu S.L., Zhong P. (2005). Shock wave interaction with laser-generated single bubbles. Phys. Rev. Lett..

[22] Goncalves E., Hoarau Y., Zeidan D. (2018). Simulation of shock-induced bubble collapse using a four-equation model. Shock Waves.

[23] Johnsen E., Colonius T. (2008). Shock-induced collapse of a gas bubble in shockwave lithotripsy. J. Acoust. Soc. Am..

[24] Goncalves da Silva E., Parnaudeau P. (2021). Numerical study of pressure loads generated by a shock-induced bubble collapse. Phys. Fluids.

[25] Cao S., Wang G., Coutier-Delgosha O., Wang K. (2020). Shock-induced bubble collapse near solid materials: effect of acoustic impedance. J. Fluid Mech..

[26] Koukas E., Papoutsakis A., Gavaises M. (2023). Numerical investigation of shock-induced bubble collapse dynamics and fluid–solid interactions during shock-wave lithotripsy. Ultrason. Sonochemistry.

[27] Reuter F., Gonzalez-Avila S.R., Mettin R., Ohl C.-D. (2017). Flow fields and vortex dynamics of bubbles collapsing near a solid boundary. Phys. Rev. Fluids.

[28] Koch M., Lauterborn W., Lechner C., Mettin R. (2023). Ring vortex dynamics following jet formation of a bubble expanding and collapsing close to a flat solid boundary visualized via dye advection in the framework of OpenFOAM. Fluids.

[29] Pezeril T., Saini G., Veysset D., Kooi S., Fidkowski P., Radovitzky R., Nelson K.A. (2011). Direct visualization of laser-driven focusing shock waves. Phys. Rev. Lett..

[30] Veysset D., Maznev A.A., Pezeril T., Kooi S., Nelson K.A. (2016). Interferometric analysis of laser-driven cylindrically focusing shock waves in a thin liquid layer. Sci. Rep..

[31] Veysset D., Gutiérrez-Hernández U., Dresselhaus-Cooper L., De Colle F., Kooi S., Nelson K.A., Quinto-Su P.A., Pezeril T. (2018). Single-bubble and multibubble cavitation in water triggered by laser-driven focusing shock waves. Phys. Rev. E.

[32] Kai Y., Lem J., Ossiander M., Meretska M.L., Sokurenko V., Kooi S.E., Capasso F., Nelson K.A., Pezeril T. (2023). High-power laser beam shaping using a metasurface for shock excitation and focusing at the microscale. Opt. Express.

[33] Liu C., Yang X., Li J., Hu Y., Zhao M., Hu C. (2024). Investigations on the shock wave induced by collapse of a toroidal bubble. Phys. Rev. E.

[34] Gutiérrez-Hernández U.J., Reese H., Ohl C.-D., Quinto-Su P.A. (2022). Bullseye focusing of cylindrical waves at a liquid–solid interface. Phys. Fluids.

[35] Gutiérrez-Hernández U.J., Reese H., Reuter F., Ohl C.-D., Quinto-Su P.A. (2023). Nano-cracks and glass carving from non-symmetrically converging shocks. Adv. Phys. Res..

[36] Chew L.W., Klaseboer E., Ohl S.-W., Khoo B.C. (2011). Interaction of two differently sized oscillating bubbles in a free field. Phys. Rev. E.

[37] Yuan F., Sankin G., Zhong P. (2011). Dynamics of tandem bubble interaction in a microfluidic channel. J. Acoust. Soc. Am..

[38] Han B., Köhler K., Jungnickel K., Mettin R., Lauterborn W., Vogel A. (2015). Dynamics of laser-induced bubble pairs. J. Fluid Mech..

[39] Cui P., Wang Q., Wang S., Zhang A. (2016). Experimental study on interaction and coalescence of synchronized multiple bubbles. Phys. Fluids.

[40] Tomita Y., Sato K. (2017). Pulsed jets driven by two interacting cavitation bubbles produced at different times. J. Fluid Mech..

[41] Luo J., Niu Z. (2019). Jet and shock wave from collapse of two cavitation bubbles. Sci. Rep..

[42] Liang W., Chen R., Zheng J., Li X., Lu F. (2021). Interaction of two approximately equal-size bubbles produced by sparks in a free field. Phys. Fluids.

[43] Mishra A., Bourquard C., Roy A., Lakkaraju R., Ghosh P., Supponen O. (2022). Flow focusing from interacting cavitation bubbles. Phys. Rev. Fluids.

[44] Fan Y., Bußmann A., Reuter F., Bao H., Adami S., Gordillo J.M., Adams N., Ohl C.-D. (2024). Amplification of supersonic microjets by resonant inertial cavitation-bubble pair. Phys. Rev. Lett..

[45] Reuter F., Mur J., Petelin J., Petkovsek R., Ohl C.-D. (2024). Shockwave velocimetry using wave-based image processing to measure anisotropic shock emission. Phys. Fluids.

[46] Mur J., Reuter F., Kocica J.J., Lokar Z., Petelin J., Agrez V., Ohl C.-D., Petkovsek R. (2022). Multi-frame multi-exposure shock wave imaging and pressure measurements. Opt. Express.

[47] Brennen C.E. (1995). https://www.ebook.de/de/product/3672751/christopher_e_brennen_cavitation_and_bubble_dynamics.html.

[48] Adams N.A., Adami S., Bogdanov V., Buhendwa A., Bußmann A., Fleischmann N., Hoppe N., Hosseini N., Kaiser J., Lunkov A., Paula T., Spaeth F., Siguenza Torres A., Wauligmann P., Winter J., Gymnich T. (2022). https://mediatum.ub.tum.de/1647482.

[49] Hoppe N., Adami S., Adams N.A. (2022). A parallel modular computing environment for three-dimensional multiresolution simulations of compressible flows. Comput. Methods Appl. Mech. Engrg..

[50] Paula T., Adami S., Adams N.A. (2023). A robust high-resolution discrete-equations method for compressible multi-phase flow with accurate interface capturing. J. Comput. Phys..

[51] Harlow F.A., Amsden A. (1971).

[52] Tomita Y., Shima A. (1977). On the behavior of a spherical bubble and the impulse pressure in a viscous compressible liquid. Bull. JSME.

[53] Dreyer W., Duderstadt F., Hantke M., Warnecke G. (2011). Bubbles in liquids with phase transition. Contin. Mech. Thermodyn..

[54] Lauer E., Hu X., Hickel S., Adams N. (2012). Numerical modelling and investigation of symmetric and asymmetric cavitation bubble dynamics. Comput. & Fluids.

[55] Koch M., Lechner C., Reuter F., Köhler K., Mettin R., Lauterborn W. (2016). Numerical modeling of laser generated cavitation bubbles with the finite volume and volume of fluid method, using OpenFOAM. Comput. & Fluids.

[56] Liang X.-X., Linz N., Freidank S., Paltauf G., Vogel A. (2022). Comprehensive analysis of spherical bubble oscillations and shock wave emission in laser-induced cavitation. J. Fluid Mech..

[57] Phan T.-H., Nguyen V.-T., Duy T.-N., Kim D.-H., Park W.-G. (2022). Influence of phase-change on the collapse and rebound stages of a single spark-generated cavitation bubble. Int. J. Heat Mass Transfer.

[58] Gottlieb S., Shu C.-W. (1998). Total variation diminishing Runge-Kutta schemes. Math. Comp..

[59] Jiang G.-S., Shu C.-W. (1996). Efficient implementation of weighted ENO schemes. J. Comput. Phys..

[60] Einfeldt B. (1988). On godunov-type methods for gas dynamics. SIAM J. Numer. Anal..

[61] Fornberg B. (1988). Generation of finite difference formulas on arbitrarily spaced grids. Math. Comp..

[62] Meng J. (2016).

[63] Toro E.F. (2009).

[64] Clain S., Diot S., Loubère R. (2011). A high-order finite volume method for systems of conservation laws—Multi-dimensional optimal order detection (MOOD). J. Comput. Phys..

[65] Harten A. (2010). Multiresolution algorithms for the numerical solution of hyperbolic conservation laws. Comm. Pure Appl. Math..

[66] Vasilyev O.V., Bowman C. (2000). Second-generation wavelet collocation method for the solution of partial differential equations. J. Comput. Phys..

[67] Shu C.-W. (1999). High-Order Methods for ComputationalPhysics.

[68] Reuter F., Deiter C., Ohl C.-D. (2022). Cavitation erosion by shockwave self-focusing of a single bubble. Ultrason. Sonochemistry.

[69] Zhang Y., Yang C., Qiang H., Zhong P. (2019). Nanosecond shock wave-induced surface acoustic waves and dynamic fracture at fluid-solid boundaries. Phys. Rev. Res..

[70] Kiyama A., Tagawa Y., Ando K., Kameda M. (2015). Effects of a water hammer and cavitation on jet formation in a test tube. J. Fluid Mech..

[71] Hawker N.A., Ventikos Y. (2012). Interaction of a strong shockwave with a gas bubble in a liquid medium: a numerical study. J. Fluid Mech..

[72] Wang Q. (2016). Local energy of a bubble system and its loss due to acoustic radiation. J. Fluid Mech..

[73] Lyu X., Pan S., Hu X., Adams N.A. (2018). Numerical investigation of homogeneous cavitation nucleation in a microchannel. Phys. Rev. Fluids.

[74] Denner F. (2021). The gilmore-NASG model to predict single-bubble cavitation in compressible liquids. Ultrason. Sonochemistry.

[75] Trummler T., Schmidt S.J., Adams N.A. (2021). Numerical investigation of non-condensable gas effect on vapor bubble collapse. Phys. Fluids.

[76] Zhao X., Ma W., Wang K. (2023). Simulating laser-fluid coupling and laser-induced cavitation using embedded boundary and level set methods. J. Comput. Phys..

[77] Fu L., Liang X.-X., Wang S., Wang S., Wang P., Zhang Z., Wang J., Vogel A., Yao C. (2023). Laser induced spherical bubble dynamics in partially confined geometry with acoustic feedback from container walls. Ultrason. Sonochemistry.

[78] Vogel A., Busch S., Parlitz U. (1996). Shock wave emission and cavitation bubble generation by picosecond and nanosecond optical breakdown in water. J. Acoust. Soc. Am..

[79] Noack J., Vogel A. (1999). Laser-induced plasma formation in water at nanosecond to femtosecond time scales: calculation of thresholds, absorption coefficients, and energy density. IEEE J. Quantum Electron..

[80] Akhatov I., Lindau O., Topolnikov A., Mettin R., Vakhitova N., Lauterborn W. (2001). Collapse and rebound of a laser-induced cavitation bubble. Phys. Fluids.

[81] Akhatov I., Vakhitova N., Topolnikov A., Zakirov K., Wolfrum B., Kurz T., Lindau O., Mettin R., Lauterborn W. (2002). Dynamics of laser-induced cavitation bubbles. Exp. Therm Fluid Sci..

[82] Bempedelis N., Ventikos Y. (2020). Energy focusing in shock-collapsed bubble arrays. J. Fluid Mech..

[83] Fuster D., Dopazo C., Hauke G. (2011). Liquid compressibility effects during the collapse of a single cavitating bubble. J. Acoust. Soc. Am..

